# A positive feed-forward loop between LncRNA-URRCC and EGFL7/P-AKT/FOXO3 signaling promotes proliferation and metastasis of clear cell renal cell carcinoma

**DOI:** 10.1186/s12943-019-0998-y

**Published:** 2019-04-05

**Authors:** Wei Zhai, Rujian Zhu, Junjie Ma, Dongkui Gong, Haimin Zhang, Jin Zhang, Yonghui Chen, Yiran Huang, Junhua Zheng, Wei Xue

**Affiliations:** 1 Department of Urology, Renji Hospital, School of Medicine in Shanghai Jiao Tong University, 160 Pujian Road, Pudong District, Shanghai, 200127 China; 2 Department of Urology, Shanghai Pudong Hospital, Fudan University Pudong Medical Center, 2800 Gongwei Road, Pudong, Shanghai, 201399 China; 3 Department of Urology, Shanghai Tenth People’s Hospital, Nanjing Medical University, Nanjing, 211166 China; 4 Department of Urology, Shanghai Tenth People’s Hospital, School of Medicine in Tongji University, Shanghai, 200072 China; 5 Department of Urology, Shanghai First People’s Hospital, School of Medicine in Shanghai Jiao Tong University, Shanghai, 200080 China

**Keywords:** Clear cell renal cell carcinoma, Long noncoding RNA, Proliferation, Invasion, EGFL7, FOXO3

## Abstract

**Background:**

The aberrant expression of long noncoding RNAs (lncRNAs) has recently emerged as key molecules in human cancers; however, whether lncRNAs are implicated in the progression of clear cell renal cell carcinoma (ccRCC) remains unclear.

**Methods:**

Candidate lncRNAs were selected using microarray analysis and quantitative real-time PCR (qRT-PCR) was performed to detect lncRNAs expression in human ccRCC tissues. Overexpression and knocking down experiments in vivo and in vitro were performed to uncover the biological roles of lncRNA-URRCC on ccRCC cell proliferation and invasion. Microarray, chromatin immunoprecipitation, Luciferase reporter assay and western blot were constructed to investigate the molecular mechanisms underlying the functions of lncRNA-URRCC.

**Results:**

The microarray analysis and qRT-PCR identified a new lncRNA, URRCC, whose expression is upregulated in RCC samples and associated with poor prognosis, leading to promote ccRCC cell proliferation and invasion. Mechanistically, URRCC enhances the expression of EGFL7 via mediating histone H3 acetylation of EGFL7 promoter, activation of P-AKT signaling, and suppressing P-AKT downstream gene, FOXO3. In return, FOXO3 could inhibit the transcription of URRCC via binding to the special region on the promoter of URRCC.

**Conclusions:**

Our data suggests that targeting this newly identified feed-back loop between LncRNA-URRCC and EGFL7/P-AKT/FOXO3 signaling may enhance the efficacy of existing therapy and potentially imparts a new avenue to develop more potent therapeutic approaches to suppress RCC progression.

**Electronic supplementary material:**

The online version of this article (10.1186/s12943-019-0998-y) contains supplementary material, which is available to authorized users.

## Introduction

Renal cell carcinoma (RCC) is one of the most aggressive human genitourinary cancers and accounts for approximately 4% of adult malignancies [1, 2]. The most common histologic subtype clear cell RCC (ccRCC) derives from the epithelial cells of the proximal renal tubule and associates with high metastatic rate and poor prognosis. Accumulating evidence has demonstrated that ccRCC resists radiotherapy and chemotherapy [3, 4]. Anti-angiogenesis drugs targeting VEGF signaling, such as multiple kinase inhibitors (Sunitinib or Pazopanib) have been recently approved and used for the treatment of advanced or metastatic ccRCC. However, the therapeutic effects are limited for a short time and the patients eventually relapse [5]. Therefore, the detailed mechanism exploring ccRCC tumorigenesis is needed to be testified.

Long non-coding RNAs (lncRNAs) represent a class of non-protein coding RNA species longer than 200 nucleotides in length [6, 7]. Functionally, lncRNAs emerge as the important players in carcinogenesis and have various biological effects such as cell apoptosis, growth, migration, invasion, metastasis and so forth [8–11]. Mechanistically, lncRNAs involve miRNA binding sites and serve as sponges to arrest miRNA function [12]. Besides, lncRNAs directly interacts with proteins to augment or attenuate their function [13]. Furthermore, lncRNAs impart their functions in new available strategies for early clinical cancer diagnosis and therapy [14–16]. In renal cancer, the aberrant expression signatures of lncRNAs have been revealed, contributing to new insights into the exploration and discovery of genitourinary malignancies [17, 18]. However, the validation of lncRNAs for clinical biomarkers and the identification of lncRNAs for molecular mechanisms in ccRCC are yet to be elucidated.

Epidermal growth factor-like domain-containing protein 7 (EGFL7), which is up-regulated in several cancers, is involved in neoplasm growth and metastasis [19–21]. Not only EGFL7 plays a key role in the promotion of hepatocellular carcinoma metastasis and glioma angiogenesis [19, 22], but also its stimulating effect on fibroblast proliferation and migration may provide a useful strategy for wound healing [23]. Furthermore, EGFL7 is reported to be epigenetic modified in gastric cancer and esophageal squamous cell carcinoma [24, 25]. Therefore, as a vital tumor-inducer, EGFL7 involving the epigenetic network in ccRCC progression holds promise for ccRCC-targeted therapy.

In this study, we focus our attention on the clinical outcomes, biological function and molecular mechanisms of a new identified lncRNA-URRCC leading to ccRCC progression. Upregulated URRCC expression is sufficiently associated with tumor size, clinical T stage, metastasis and reduced overall survival of ccRCC patients. Mechanistically, histone H3 acetylation of EGFL7 promoter caused by URRCC could increase AKT signaling pathway and suppress P-AKT downstream FOXO3 signaling, enhancing cell proliferation and invasion in vitro and in vivo. Interestingly, FOXO3 could in turn downregulate URRCC expression via directly binding to its promoter region, thus forming a novel feedback loop which could provide a promising therapeutic target for ccRCC.

## Materials and methods

### Microarray analysis

Microarray analysis for the expression of lncRNAs was performed by Shanghai Gminix Biological Information Company (Shanghai, China), applying a pipeline as previously described [26, 27] to identify the probe sets uniquely mapped to lncRNAs from the Affymetrix array, in which special way can evaluate the lncRNA expressions in RCC gene expression data. The accession numbers for the microarray data are Gene Expression Omnibus database GEO: GSE46699 and GSE53757 [28, 29]. Gene expression profiles of the 786-O cells with or without URRCC downregulation were determined using Human Cancer Focus mRNA PCR Array following the manufacturer’s instructions. Microarray experiments were performed by KangChen Bio-tech, Shanghai, China. The differentially expressed genes with statistical significance were identified using volcano plot filtering. The threshold we used to screen upregulated or downregulated genes is fold change > = 2.0 and a *p*-value <= 0.05.

### Clinical samples

A total of 116 tumor samples and paired non-cancerous renal tissues were obtained from patients who underwent partial or radical nephrectomy in Department of Urology, Shanghai Tenth People’s Hospital, Tongji University (Shanghai, China). The fresh tissues were frozen in liquid nitrogen to protect the protein or RNA away from degradation. The use of human tissues was approved by the ethics committee of Shanghai Tenth People’s Hospital.

### Cell cultures

The human RCC cell lines A498, 786-O, CaKi-1, ACHN, 769-P and OSRC-2 and human normal renal tubular epithelial cell line HK-2 were obtained from Cell Bank of the Chinese Academy of Sciences (Shanghai, China). A498 was cultured in Dulbecco’s Modified Eagle’s Medium (DMEM, Gibco, USA) plus 10% Fetal Bovine Serum (FBS, Hyclone, USA) with 1% Penicillin/Streptomycin (P/S, Gibco, USA). 786-O, CaKi-1, ACHN, 769-P and OSRC-2 cells were cultured in RMPI 1640 (Gibco, USA) plus 10% Fetal Bovine Serum (FBS, Hyclone, USA) with 1% Penicillin/Streptomycin (P/S, Gibco, USA). HK-2 cells were cultured in Keratinocyte Medium (KM, ScienCell, USA) plus 1% Keratinocyte Growth Supplement (KGS, ScienCell, USA) with 1% Penicillin/Streptomycin (P/S, ScienCell, USA). All cells described above were cultured at 37° in 5% CO_2_.

### Cell transfection and vector construction

According to the manufacturer’s protocol, short interfering RNA (siRNA) si-EGFL7/si-FOXO3/si-NC (IBS Solutions Co. Ltd., China) were transiently transfected in A498 cells using Lipofectamine 3000 (Invitrogen, USA) at a final concentration of 100 nM. Lentiviral sh-control/sh-URRCC and oe-URRCC/oe-FOXO3 were purchased from Shanghai Integrated Biotech Solutions Co., Ltd. Lentiviral infection was performed as previously described [30, 31]. In order to avoid the off-target effects of shRNA, we designed two different shRNAs for lncRNA-URRCC and two different siRNAs for EGFL7 and FOXO3. The sequences of oligonucleotides used in this experiment were listed in Additional file 1: Table S1 and Additional file 2: Table S2.

### Fluorescent in situ hybridization (FISH)

FISH was performed as previously described [14]. It is a powerful technique that uses non-toxic fluorescent DNA probes to target any given sequence within a nucleus, resulting in colored signals that are detected with a fluorescence microscope and was performed at Biosense Co. Ltd. Paraffin-embedded tissue blocks were retrospectively retrieved from renal cancer patients. Quantum dot fluorescent in situ hybridization (QD-FISH) was performed to detect the presence of URRCC using a digoxin-labeled oligonucleotide probe indirectly labeled with digoxin-antibody-conjugated quantum dots.

### Isolation of cytoplasmic and nuclear RNA

Cytoplasmic and nuclear RNA were isolated and purified using the Cytoplasmic & Nuclear RNA Purification Kit (Norgen, Belmont, CA) according to the manufacturer’s instructions.

### RCC cell proliferation assay

RCC cells were seeded in 24-well plates (3000 cells/well) and cultured for 0, 2, 4, or 6 days. Cells were harvested and cell numbers were calculated using MTT agent. DMSO was used as the control. We added 250 μl of 5 mg/ml MTT to each well, incubated for 2 h in incubator at 37 °C, removed the media and added 150 μl DMSO. We then covered with tinfoil and agitated cells on orbital shaker for 15 min and then read the absorbance at 570 nm.

### RCC cell invasion assay

The invasive capability of RCC cells was determined by the Transwell assay. The upper chamber of Transwll was pre-covered with Matrigel (BD356230, CORNING, USA). RCC cells were harvested and seeded with serum-free DMEM into the upper chambers at 5 × 10^4^ cells/well, and the bottom chambers contained DMEM with 10% FBS, and then transwells incubated for 24 or 36 h at 37 °C. Following incubation, the invasive cells attached to the lower surface of the membrane were fixed by 4% paraformaldehyde and stained with 1% toluidine blue. Cell numbers were counted in five randomly chosen microscopic fields (100×) per membrane.

### Xenograft subcutaneous implantation and tail vein implantation

Six-week-old male BALB/c nude mice were purchased from ShanghaiSipper-BK laboratory animal Company (Shanghai, China). After 5 days of adapting to the environment, a total of 1 × 10^6^ A498 cells (stably transfected with sh-URRCC and sh-control) and OSRC-2 cells (stably transfected with oe-URRCC and mock) were injected hypodermically into the right armpit or tail vein of each mouse, respectively (*n* = 8 mice/group). Cells were also transduced with luciferase for the non-invasive in vivo imaging system (IVIS) (NightOWL II, LB983, Berthold Technologies, Germany) that was performed once a week. The tumor volume and the weight of each mouse were measured per week. The mice were killed on the 60th days after injection. We also measured and recorded the weight of each tumor. All animal studies were approved by the Institutional Animal Care and Use Committee of the Shanghai Tenth People’s hospital.

### RNA isolation and quantitative real-time PCR (qRT-PCR)

Total RNA was extracted from frozen tissues or cells using Trizol reagent (Invitrogen, USA) according to the manufacturer’s instructions. Reverse transcription was performed using a PrimeScript RT reagent kit (TaKaRa, Japan), and qRT–PCR was performed with KAPA SYBR FAST qPCR Kit (Kapa Biosystems, USA) using a 7900HT Fast Real-Time PCR System (Applied Biosystems, Japan). The mRNA and lncRNA levels were normalized against β-actin in cell and tissue lysates. The miRNA levels were normalized against U6. Data were analyzed using the 2^-ΔΔCt^ method. The primer sequences were listed in the Additional file 1: Table S1.

### Western blot (WB)

Protein extracts were separated from cells or human tissues by RIPA buffer containing protease inhibitors. 30 μg of protein extracts were loaded to 8–10% sodium dodecylsulfate–polyacrylamide gel electrophoresis gels and transferred onto nitrocellulose membranes. The membranes were hybridized with a primary antibody at 4 °C overnight, and incubated with a secondary antibody in 1 h at room temperature. The expression of β-actin was used as loading control. The intensity of the fluorescence was scanned by the Odyssey scanner (LI-COR Biosciences, USA). The information of antibodies was listed as follow: EGFL7 (1:500, proteintech, 19,291–1-AP), AKT (1:10000, Abcam, ab179463), P-AKT (1:1000, Abcam, ab131443), FOXO3 (1:500, Abcam, ab12162), H3K27ac (1:1000, Abcam, ab4729), H3 (1:1000, Abcam, ab1791), β-actin (11,000, Abcam, ab8226).

### Immunohistochemistry (IHC)

Immunohistochemistry for the target molecules was performed on paraffin sections using a primary antibody against EGFL7 (1:50, proteintech, 19,291–1-AP), FOXO3 (1:500, Abcam, ab12162), P-AKT (1:50, Abcam, ab131443), Ki67 (1:500, Abcam, ab92742) and the proteins in situ were visualized with 3, 3-diaminobenzidine. For histological scoring, 3 high power fields (magnification, × 400) were randomly selected from renal cancer tissues and normal renal tissues. The reactivity degree was assessed by at least two pathologists without knowledge of the clinicopathological features of the tumors. The degree of positivity was initially classified according to scoring both the proportion of positive staining tumor cells and the staining intensities. Scores representing the proportion of positively stained tumor cells were graded as: 0 (< 10%); 1 (11–25%); 2 (26–50%); 3 (51–75%) and 4 (> 75%). The intensity of staining was determined as: 0 (no staining); 1 (weak staining = light yellow); 2 (moderate staining = yellow brown); and 3 (strong staining = brown). The staining index (SI) was calculated as the product of staining intensity × percentage of positive tumor cells, resulting in scores of 0, 1, 2, 3, 4, 6, 8, 9 and 12. Only cells with clear tumor cell morphology were scored.

### Chromatin immunoprecipitation assay (ChIP)

EZ ChIP™ Chromatin Immunoprecipitation Kit (Millipore, Bedford, MA, USA) and the EpiQuik Tissue Acetyl-Histone H3 and H4 CHIP Kit (Epigentek Group Inc., NY, USA) were used to perform ChIP assays. In brief, cells were crosslinked with 4% formaldehyde for 10 min followed by cell collection and sonication with a predetermined power to yield genomic DNA fragments of 300–1000 bp long. The chromatin was immunoprecipitated using an anti-acetyl-histone H3 or anti-acetyl-histone H4 antibody, or anti-FOXO3 antibody. Normal mouse IgG or normal rabbit IgG were used as the negative control. qRT-PCR was conducted using KAPA SYBR FAST qPCR Kit (Kapa Biosystems, USA). PCR products were analyzed by agarose gel electrophoresis. Specific primer sequences are listed in Additional file 1: Table S1.

### Luciferase reporter assay

Cells were co-transfected with psiCHECK2 dual-luciferase reporter and pcDNA3.1-URRCC-promoter (wt or mt) or pcDNA3.1-FOXO3 using Lipofectamine 3000 (Invitrogen, USA). All groups were run in triplicate in 48-well plates. Luciferase activity was measured by Dual-Luciferase Assay (Promega, Madison, WI, USA) according to the manufacturer’s manual after 48 h of transfection. Renilla luciferase activity was normalized against Firefly luciferase activity.

### Statistical analysis

The preprocessed level 3 RNA-seq data and corresponding clinical information of clear cell renal cell carcinoma patients were collected from The Cancer Genome Atlas (TCGA) database (http:// cancergenome.nih.gov/). All statistical analysis was done using IBM SPSS Statistics (Version 20.0, IBM) and Graphpad Prism V6 (GraphPad Software, Inc., 2012). Student’s t-test, one-way ANOVA, LSD-t test, Pearson chi-square test, Log-rank test, linear regression, and Cox Regression Analyses were performed as indicated. All data was considered significantly when *p<0.05.

## Results

### URRCC is a novel lncRNA involved in renal malignant transformation

To search for the potential lncRNAs involved in ccRCC progression, we systematically analyzed the dis-regulated lncRNAs expression profiles of ccRCC tissues versus matched normal kidney tissues using two published GEO DataSets (GSE46699 and GSE53757). The detailed program was shown in Additional file 3: Figure S1A.

**Fig. 1:**
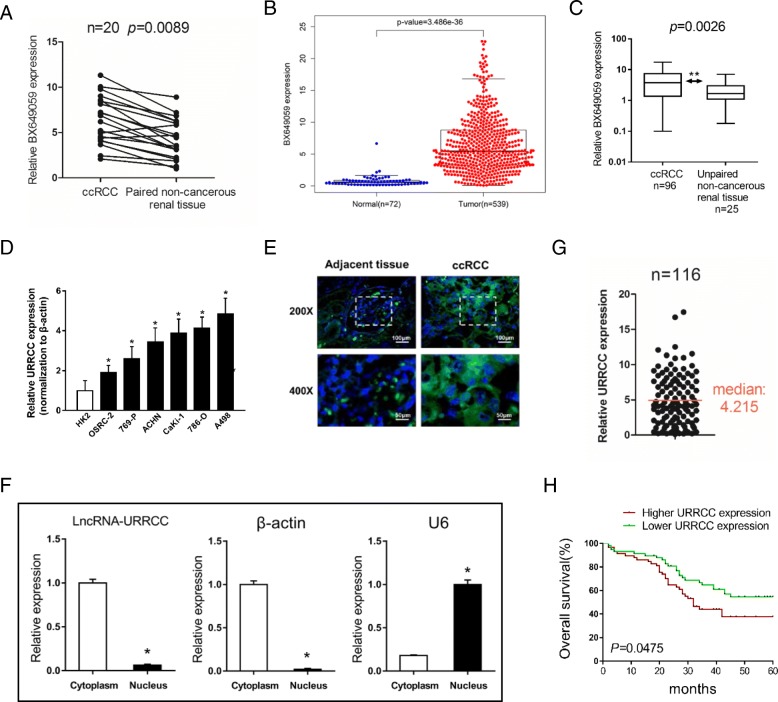
URRCC is a novel lncRNA involved in renal malignant transformation. **a**: Comparison of BX649059 expression in 20 paired renal cancer tissues and adjacent non-cancer tissues via qRT-PCR. **b**: BX649059 expression in ccRCC tissues and normal tissues from TCGA KIRC dataset. **c**: BX649059 expression was determined via qRT-PCR in 96 renal cancer tissues and 25 unpaired non-cancerous renal tissues. **d**: URRCC expression in a series of RCC cell lines (OSRC-2, 769-P, ACHN, Caki-1, 786-O and A498) and human normal renal tubular epithelial cell line HK-2. **e**: RNA FISH analysis of URRCC (green) in adjacent tissues and ccRCC tissues while the nucleuses were stained with DAPI (blue). **f**: qRT-PCR for URRCC, U6 and β-actin from RNA extracted from cytoplasmic and nuclear fractions. **g**: Of the enrolled 116 cases, the relative URRCC mRNA levels were tested in convenience of the logistic regression analysis. **h**: Kaplan–Meier analyses of the correlations between URRCC expression level and overall survival of 116 patients with RCC. The median expression level was used as the cutoff

We computed the intersection between the result sets of these two datasets through heatmap analysis and focused on 3 potential lncRNAs (AK026225, BX649059 and AK055783) whose expression increased among top 50 lncRNAs that up-regulated in ccRCC tissues compared to paired normal renal tissues (Additional file 3: Figure S1B and C). The expression of these 3 lncRNAs was validated using qRT-PCR analysis and the outcome showed that only BX649059 expression was dramatically higher in 20 ccRCC tissues compared to paired noncancerous renal tissues (Fig. 1a), which was consistent with the result of datasets and the analysis of ccRCC patients from TCGA KIRC dataset (Fig. 1b and Additional file 4: Table S3). However, the other two lncRNAs (AK026225 and AK055783) seemed to be no differences between ccRCC tissues and paired noncancerous renal tissues (Additional file 3: Figure S1D). Moreover, the transcript level of BX649059 was reproducibly upregulated in 96 ccRCC tissues compared to 25 unpaired normal renal tissues (Fig. 1c). Therefore, we named BX649059 as LncRNA-URRCC (up-regulation in clear cell renal cell carcinoma). In addition, the qRT-PCR analysis revealed remarkably higher URRCC expression in a series of human renal cancer cell lines than that in human normal renal tubular epithelial cell line, HK2 cells (Fig. 1d). By contrast, such a change was not observed in the expression of the other two lncRNAs (AK026225 and AK055783) (data no shown).

Using Ensemble software, we further confirmed the character of URRCC was from clone DKFZp779P0730, position within chromosome 12: 100,623,715-100,628,286 (Additional file 3: Figure S1E). The full sequence of URRCC, with transcript length 3967 bp, was presented in Additional file 3: Figure S1F. Furthermore, RNA fluorescence in situ hybridization (FISH) on ccRCC tissues detected that URRCC (green) was mainly located in the cytoplasm while the nucleuses were stained with DAPI (blue). Subcellular fraction assay also found that URRCC was mainly located in the cytoplasm, consistent with online software lncLocator (http://www.csbio.sjtu.edu.cn/bioinf/lncLocator/) predicting the location of URRCC (Fig. 1e, f and Additional file 3: Figure S1G).

### URRCC associates with ccRCC poor prognosis

To further explore the relationship between URRCC expression and the clinicopathological characteristics of 116 patients with ccRCC, we analyzed URRCC expression in ccRCC tissues. We ranked URRCC mRNA level and selected the median (4.215) of the whole set of data as the cutoff point between the high URRCC group and the low URRCC group (Fig. 1g). Clinicopathological analysis confirmed that high level of URRCC in tumors was associated with tumor size, T stage and metastasis (Additional file 5: Table S4). In the univariate analysis, high URRCC expression in tumors (hazard ratio, HR = 2.59; 95% confidence interval, CI = 1.46–4.33; *p* = 0.001), Tumor size (HR = 1.59; 95%CI = 1.44–2.74; *p* = 0.035), T stage (HR = 1.42; 95%CI = 1.25–2.68; *p* = 0.038) and metastasis (HR = 1.47; 95%CI = 1.05–1.86; *p* = 0.026) was remarkably correlated with overall survival (Additional file 6: Table S5). Multivariate analysis showed that a higher URRCC expression (HR = 2.44; 95%CI = 1.36–4.40; *p* = 0.003) were significantly associated with overall survival (Additional file 6: Table S5). In addition, Kaplan-Meier analysis in the 116 patients with ccRCC demonstrated that high URRCC expression level in ccRCC tissues dramatically correlated with a reduction in 5 years overall survival rates (Fig. 1h).

### URRCC promotes the development of progression in ccRCC cell lines

**Fig. 2:**
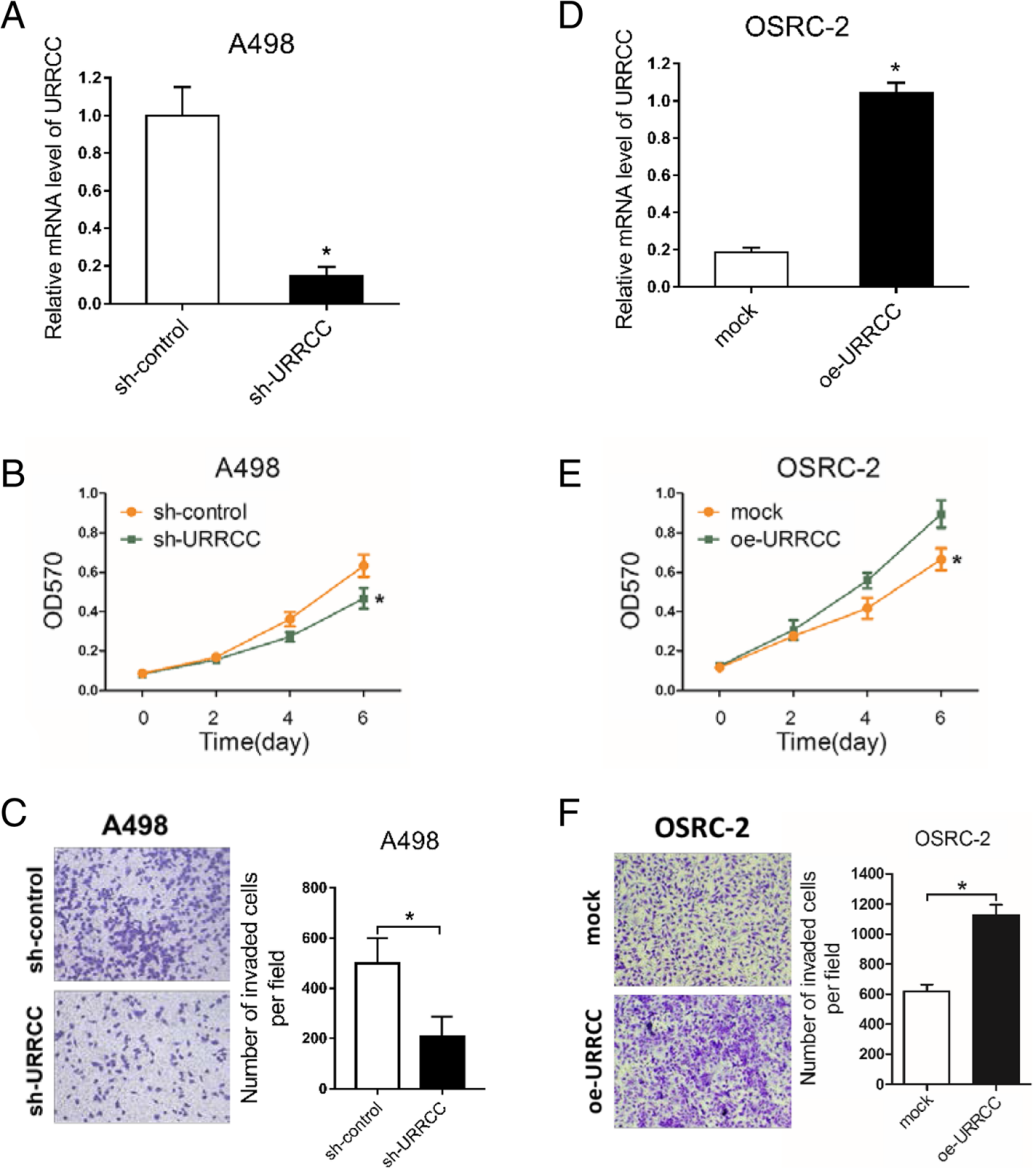
URRCC promotes the development of progression in ccRCC cell lines. **a** and **d**: qRT-PCR assays for the URRCC mRNA level in indicated ccRCC cell clones. **b** and **e**: MTT assays after transfection of sh-URRCC or oe-URRCC compared with sh-control or mock in A498 (**b**) and OSRC-2 (**e**) cells. **c** and **f**: Representative images and the numbers of invasive cells per high-power field reduced by the transfection of sh-URRCC or oe-URRCC in A498 (**c**) and OSRC-2 (**f**) compared to sh-control or mock groups. Black scale bar represents 100 μm

The result above demonstrated that URRCC overexpressed in ccRCC tissues and potentially used as diagnostic or prognostic markers. To ascertain whether URRCC could impact RCC progression, we chose the shRNA with the best interference from two shRNAs, which were confirmed in 786-O and OSRC-2 cells (Additional file 7: Figure S2A and B). Preliminary experiments showed that knocking down the expression of URRCC could inhibit the proliferation and invasion of 786-O cells (Additional file 7: Figure S2C and D). Then we established to knock down URRCC with sh-URRCC in human A498 cells. As shown in Fig. 2a and b, knocked-down URRCC dramatically decreased the growth speed of A498 cells, when compared with control cells through MTT assay. In addition, transwell invasion assay also demonstrated that sh-URRCC attenuated invasive capability of A498 cells than that of control cells (Fig. 2c). The effect of sh-URRCC was also confirmed in 786-O cells (Additional file 7: Figure S2C and D).

Conversely, we examined the tumor-inducing phenotype in OSRC-2 cells with overexpression of URRCC (oe-URRCC) (Fig. 2d). As expected, upregulating URRCC expression obviously enhanced proliferation of OSRC-2 cells compared with the mock group via MTT assay (Fig. 2e). Consistently, overexpressed URRCC also induced invasion of OSRC-2 cells through transwell invasion assay (Fig. 2f).

Together, results from Fig. 2 substantiated that URRCC could promote cell proliferation and invasion in ccRCC cells.

### URRCC enhances the development of progression in ccRCC in vivo

**Fig. 3:**
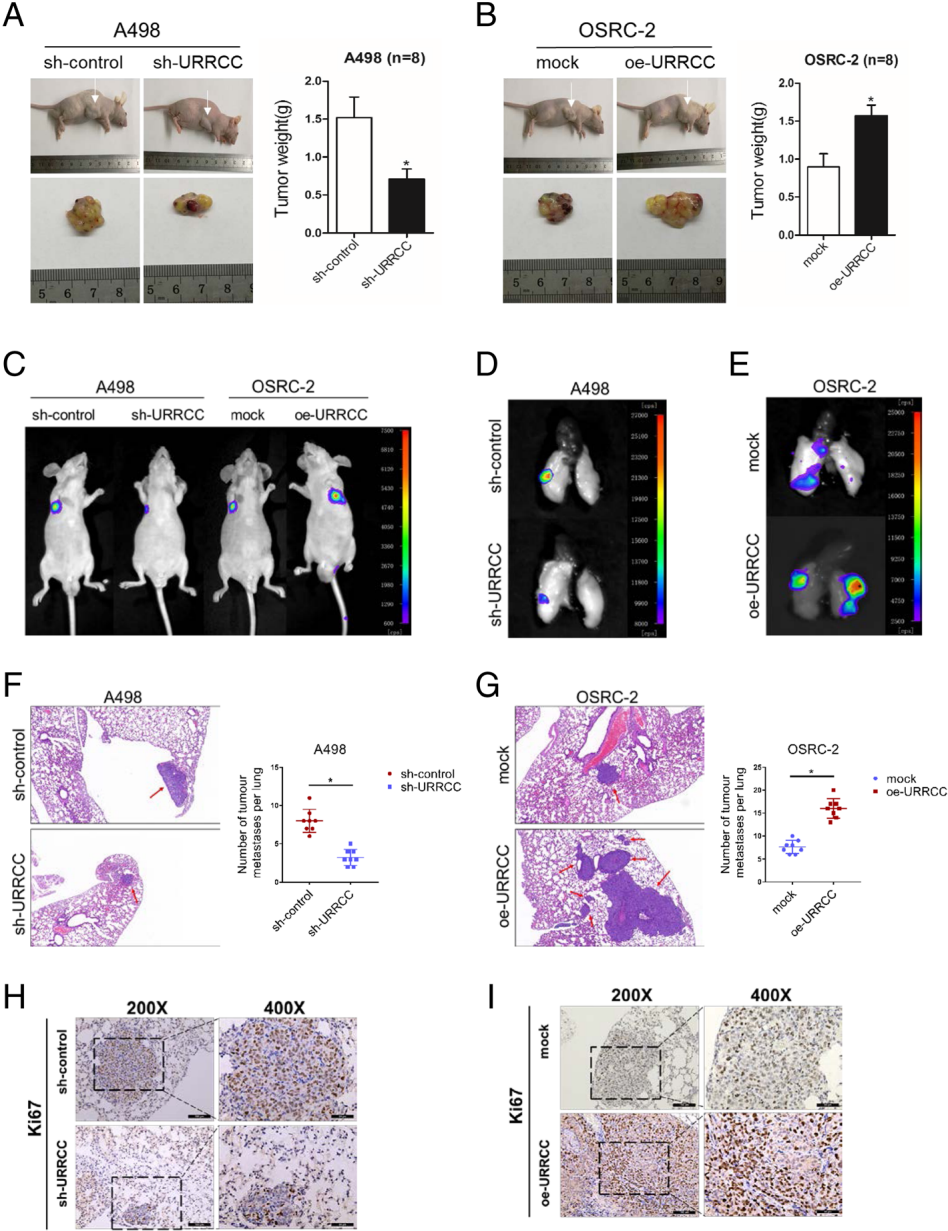
URRCC enhances the development of progression in ccRCC in vivo*.* **a**: The in vivo effect of URRCC was evaluated in xenograft mouse models bearing tumors originating from A498 cells stably transfected with sh-URRCC or sh-control, *n* = 8/group; Weights of the xenografts were shown in the right. **b**: The in vivo effect of URRCC was evaluated in xenograft mouse models bearing tumors originating from OSRC-2 cells stably transfected with oe-URRCC or mock, n = 8/group; Weights of the xenografts were shown in the right. **c**: Representative images of mice in each group at 4 weeks after tail vein injections of A498 (sh-control or sh-URRCC infected) or OSRC-2 (mock or oe-URRCC infected), n = 8/group. **d** and **e**: Representative images of pulmonary metastasis in each group at 8 weeks after tail vein injections. **f** and **g**: Representative microscopic images of pulmonary metastatic lesions at 8 weeks after tail vein injection of indicated ccRCC (left). Red arrows indicate lung metastatic tumors (100×). The number of lung metastatic tumors in each group (n = 8) was calculated (right). **h** and **i**: Representative Ki67 IHC staining of pulmonary metastasis from sh-control, sh-URRCC, mock, and oe-URRCC groups (200×, 400×)

The entire above in vitro data demonstrated that URRCC might play a positive role to enhance ccRCC cell proliferation and invasion. We next explored the proliferative role of URRCC in vivo. The A498/sh-URRCC cells as well as their corresponding control group cells were inoculated into the right flank of nude mice. As Fig. 3a showed, URRCC knocked-down effectively inhibited tumor proliferation, as revealed by measuring tumor mass. On the contrary, stable transfection of overexpressing URRCC (oe-URRCC) into OSRC-2 cells resulted in dramatically induced tumor growth (Fig. 3b).

We next generated both cells labeled with firefly luciferase and then injected into tail vein of nude mice to monitor tumor metastasis. A dramatic reduction of metastatic luciferase expression in lungs of A498/sh-URRCC mice was detected by In Vivo Imaging Systems (IVIS) compared with sh-control group (Fig. 3c and d). HE analysis also showed a decrease of pulmonary metastasis in A498/sh-URRCC group compared with sh-control group (Fig. 3f). Contrarily, URRCC overexpression yielded an enhanced pulmonary metastasis of OSRC-2 cells in vivo (Fig. 3c, e and g). In addition, IHC demonstrated the decreased expression of Ki67 from sh-URRCC group compared with sh-control group, while the increased expression of Ki67 from oe-URRCC group compared with the mock group in pulmonary metastasis (Fig. 3h and i).

Together, results from Fig. 3 demonstrated that URRCC functioned as a critical tumor enhancer in vivo by promoting tumor proliferation and metastatic colonization.

### URRCC enhances EGFL7 level by mediating histone H3 acetylation of EGFL7 promoter

**Fig. 4:**
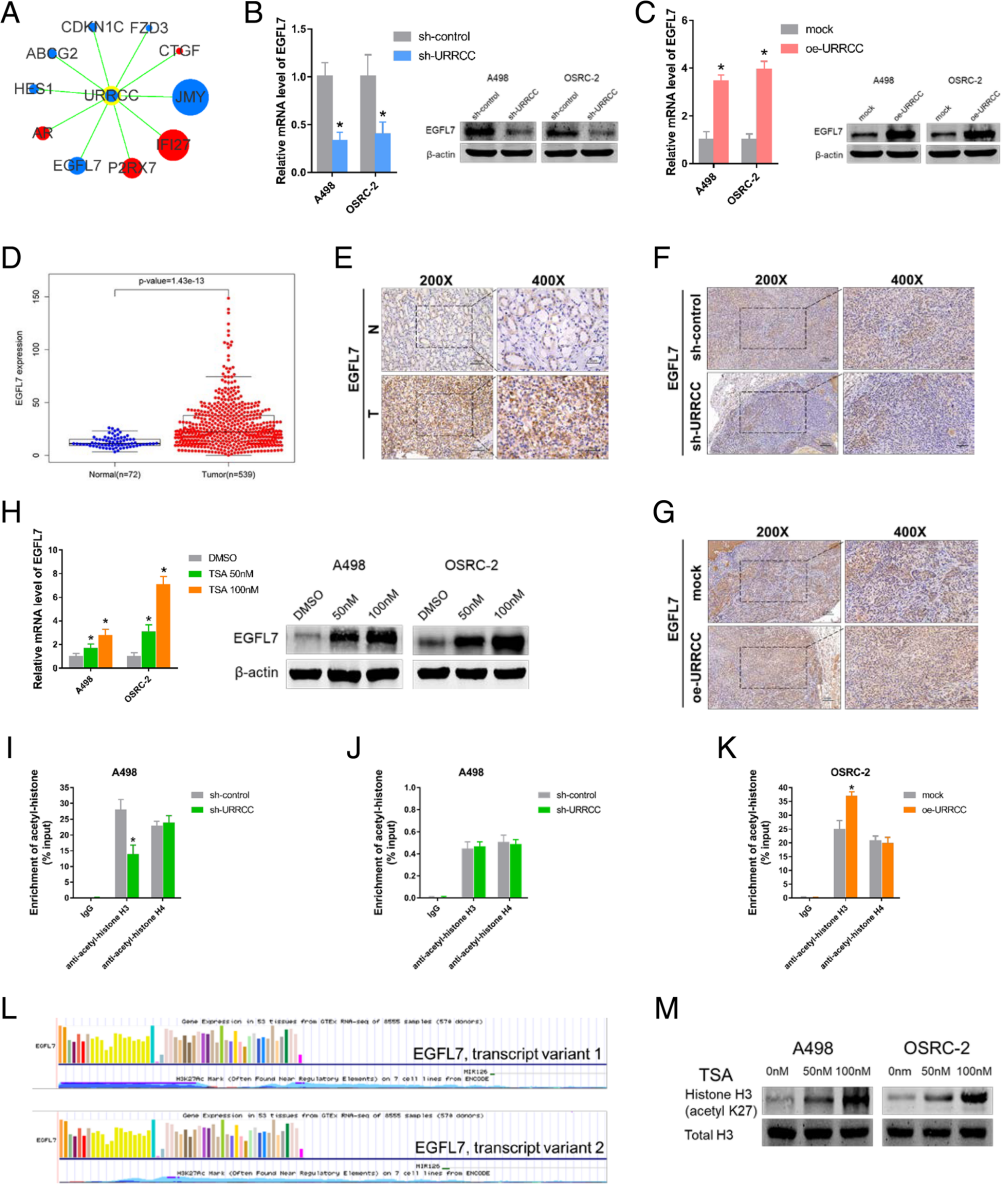
URRCC enhances EGFL7 level by mediating histone H3 acetylation of EGFL7 promoter. **a**: URRCC subnetwork according to the result of Target mRNA PCR Array. Genes colored in blue are down-regulated genes after that cells were transfected with sh-URRCC. Genes colored in red are up-regulated genes after that cells were transfected with sh-URRCC. The size of the gene round represents the fold change. **b**: The mRNA and protein levels of EGFL7 were detected by qRT-PCR and WB assays in A498 and OSRC-2 cells after transfection with sh-control or sh-URRCC. **c**: The mRNA and protein levels of EGFL7 were detected by qRT-PCR and WB assays in A498 and OSRC-2 cells after transfection with mock or oe-URRCC. **d**: EGFL7 expression in ccRCC and normal samples from TCGA KIRC dataset. **e**: Representative EGFL7 IHC staining of ccRCC tissues compared to paired normal renal tissues (200×, 400×). Black scale bar represents 100 μm (200×) or 50 μm (400×). **f** and **g**: Representative EGFL7 IHC staining of xenograft tumors from sh-control, sh-URRCC, mock, and oe-URRCC groups (200×, 400×). Black scale bar represents 100 μm (200×) or 50 μm (400×). **h**: qRT-PCR and WB analysis of EGFL7 in A498 and OSRC-2 cells treated with DMSO or trichostatin A (TSA) (50 nM or 100 nM) for 72 h (*n* = 3). **i**: ChIP analyses of A498 cells transfected with sh-control or sh-URRCC were conducted on the EGFL7 promoter regions using anti-acetyl-histone H3 and anti-acetyl-histone H4. Enrichment was determined relative to input controls. The data are the means ± standard deviations of three independent biological replicates. **j**: ChIP analyses of A498 cells transfected with sh-control or sh-URRCC were conducted on the GAPDH promoter regions using anti-acetyl-histone H3 and anti-acetyl-histone H4. Enrichment was determined relative to input controls. **k**: ChIP analyses of OSRC-2 cells transfected with mock or oe-URRCC were conducted on the EGFL7 promoter regions using anti-acetyl-histone H3 and anti-acetyl-histone H4. Enrichment was determined relative to input controls. The data are the means ± standard deviations of three independent biological replicates. **l**: EGFL7 promoter region was enriched with H3K27Ac histone mark presented with UCSC data. **m**: WB analysis of H3K27ac and total H3 in A498 and OSRC-2 cells treated with DMSO or TSA, β-actin was used as a loading control

Recently, lncRNA has been reported to regulate mRNA and was independent of functioning as competing endogenous RNAs (ceRNA), although the lncRNA was mainly located in cytoplasm [14]. To investigate the potential gene related with URRCC-driven proliferative and metastatic phenotype in ccRCC, we used “Human Cancer Focus mRNA PCR Array” from KangCheng Bio-tech Inc. to compare dysregulated mRNAs that shown more than 2 fold change difference in expression between sh-control and sh-URRCC in the treated A498 cells (Additional file 8: Figure S3A, Additional file 9: Table S6). Volcano plots indicated six down-regulated mRNAs and four up-regulated mRNAs (Fig. 4a and Additional file 8: Figure S3B). We focused on genes that have the same changing trend with URRCC. Among them, JMY and EGFL7 were markedly decreased by knocking down URRCC at mRNA expression (Fig. 4a). We chose EGFL7 as our candidate for next experiments due to the tumor suppressor role of JMY (Additional file 8: Figure S3C and D, Additional file 10: Table S7 and Additional file 11: Table S8) in ccRCC. qRT-PCR and WB analysis revealed that sh-URRCC dramatically decreased the mRNA and protein levels of EGFL7 in A498 and OSRC-2 cells (Fig. 4b), whereas upregulated mRNA and protein levels of EGFL7 were identified in oe-URRCC group of both cells (Fig. 4c). Meanwhile, qRT-PCR assays showed there is a positive correlation between URRCC and EGFL7 in mRNA level in cell lines (Additional file 8: Figure S3E). In addition, The TCGA KIRC dataset analysis and IHC analysis substantiated that EGFL7 mRNA levels and protein levels were much higher in the ccRCC tissues than their noncancerous tissues (Fig. 4d and e, Additional file 12: Table S9). Moreover, subcutaneous xenograft tumors from sh-URRCC group demonstrated a decrease in EGFL7 expression by IHC compared with those from the sh-control group, while subcutaneous xenograft tumors from oe-URRCC group exhibited an increase in EGFL7 expression by IHC compared with those from the mock group (Fig. 4f and g).

To investigate the mechanism responsible for the upregulation of EGFL7 in ccRCC, we probed for EGFL7 could be regulated by inhibitors of histone deacetylases according to previous studies revealing that the histone acetylation was the important regulation approach for EGFL7 [25]. qRT-PCR and WB assays indicated that EGFL7 was upregulated via the histone deacetylase inhibitor trichostatin A (TSA) in A498 and OSRC-2 cells (Fig. 4h). Meanwhile, ChIP assay confirmed that sh-URRCC caused a significant decreased level of histone H3 acetylation, but not histone H4 across EGFL7 promoter region (− 2000 to + 50 bp relative to Transcriptional start site (TSS)) in A498 cells (Fig. 4i). Additional ChIP assay indicated that sh-URRCC could not affect the H3 and H4 acetylation of GAPDH promoter in A498 cells (Fig. 4j). Conversely, oe-URRCC increased histone H3 acetylation level across EGFL7 promoter region in OSRC-2 cells (− 2000 to + 50 bp relative to TSS) (Fig. 4k). To further investigate the mechanism that why EGFL7 expression was regulated by histone H3 acetylation, we explored the potential histone acetylation sites from the promoter of EGFL7 via UCSC browser. Interestingly, aberrant enrichment of the H3K27ac histone mark were found across the promoter region of EGFL7 (Fig. 4l). Consistently, we found TSA could increase expression of H3K27ac in protein level in A498 and OSRC-2 cells, which may explain why H4 acetylation was not affected (Fig. 4m). Moreover, ChIP assay showed that TSA could reverse the down-regulation of EGFL7 caused by sh-URRCC both on acetylation and expression of EGFL7 (Additional file 8: Figure S3F-H).

Taken together, all results above illustrated that URRCC enhanced EGFL7 expression by mediating histone H3 acetylation across EGFL7 promoter region.

### URRCC is associated with EGFL7/P-AKT/FOXO3 signaling pathway

**Fig. 5:**
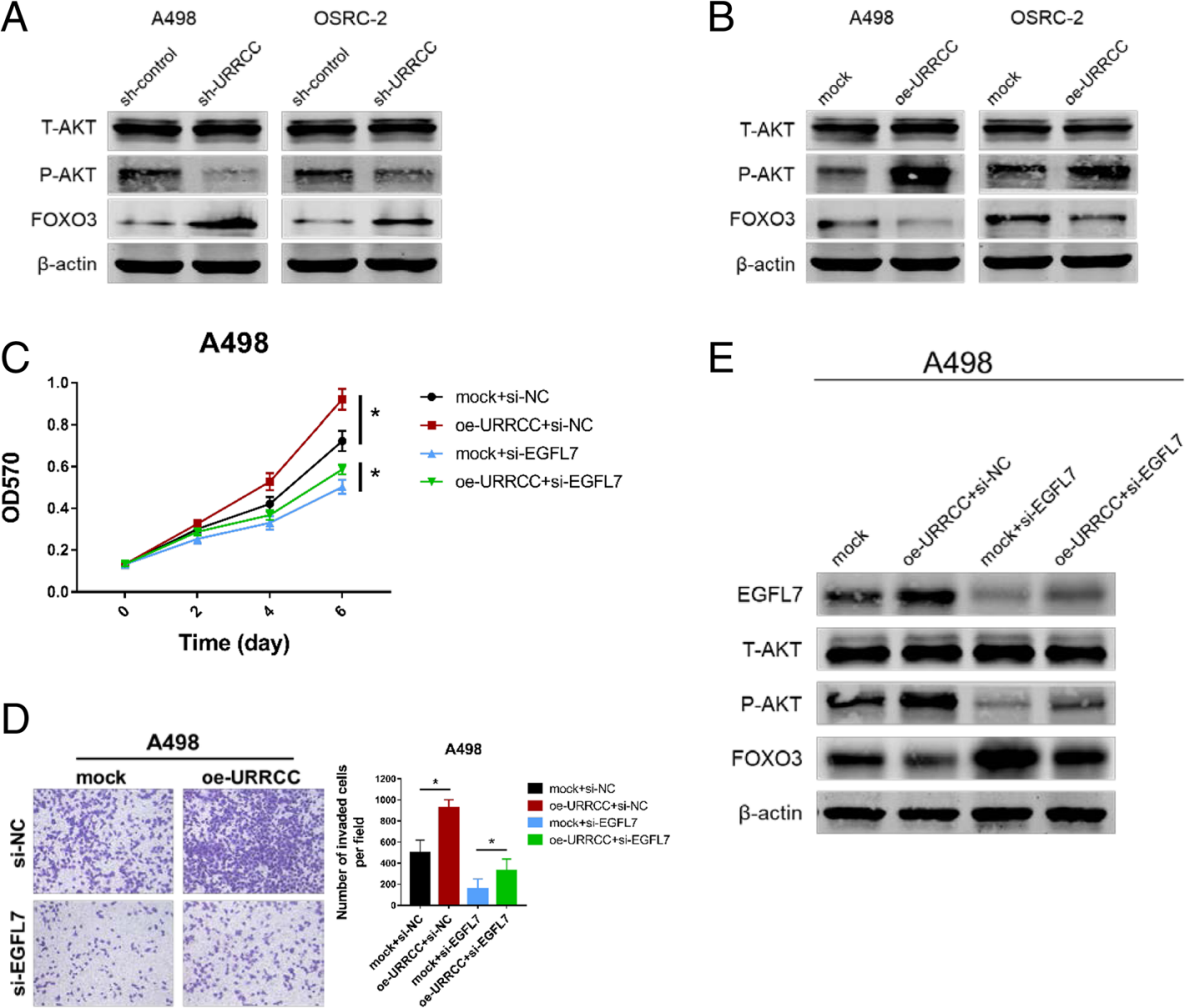
URRCC is associated with EGFL7/P-AKT/FOXO3 signaling pathway. **a** and **b**: WB analysis of T-AKT, P-AKT, and FOXO3 in URRCC-overexpressing or URRCC-inhibited A498 and OSRC-2 cells compared with sh-control or mock group, β-actin was used as a loading control. **c**: CCK8 assays of A498 cells transfected with mock/oe-URRCC and si-NC/si-EGFL7. **d**: Representative images and the numbers of invasive cells per high-power field reduced after the transfection with mock/oe-URRCC and si-NC/si-EGFL7 in A498 cells. **e**: WB analysis for EGFL7, T-AKT, P-AKT, and FOXO3 protein levels of A498 cells transfected with mock/oe-URRCC and si-NC/si-EGFL7. β-actin was used as a loading control

Aberrant AKT signaling pathway played an important role in ccRCC and our group constantly concentrated on the studies of AKT signaling pathway [30, 32]. To investigate whether URRCC could regulate AKT signaling pathway in ccRCC, we performed WB assays and the result indicated that sh-URRCC could blunt P-AKT signaling pathway in both A498 and OSRC-2 cell lines (Fig. 5a). Concomitant with the decrease of P-AKT, we observed an increase of FOXO3 (Fig. 5a), which was reported in previous studies that downregulation of FOXO3 expression is due to increased AKT activity [33–35]. Contrarily, oe-URRCC could enhance EGFL7/P-AKT signaling pathway in both cell lines while decrease the FOXO3 expression (Fig. 5b). Furthermore, we identified that the phenotypes caused by oe-URRCC could be partly rescued by si-EGFL7 using CCK8 and invasion assays in A498 cells (Fig. 5c and d). Similarly, the increase of EGFL7 and the decrease of FOXO3 caused by oe-URRCC also could be rescued by si-EGFL7 through WB assays in A498 cells (Fig. 5e). IHC of subcutaneous xenograft tumors showed a decrease of P-AKT expression from the sh-URRCC group compared with the sh-control group, while an increase of P-AKT expression from the oe-URRCC group compared with the mock group (Additional file 8: Figure S3I).

Altogether, our data suggested that URRCC could enhance renal cancer cell proliferation and invasion through EGFL7/P-AKT/FOXO3 signaling.

### FOXO3 inhibits URRCC transcriptional level via binding to its promoter region

**Fig. 6:**
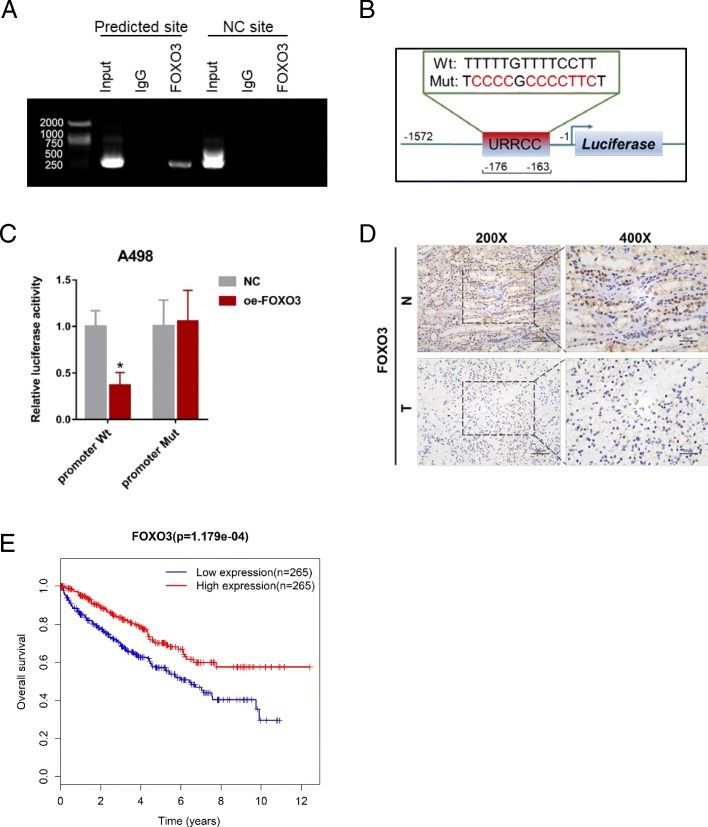
FOXO3 inhibits URRCC level via binding to its promoter region. **a**: ChIP assay showing FOXO3 can bind to potential binding site on the URRCC promoter. **b**: A schematic illustration of FOXO3 binding element mutation on the URRCC promoter. **c**:Relative luciferase activity of the indicated promoter vectors in A498 cells transfected with Renilla luciferase plasmids. **d**: Representative FOXO3 IHC staining of ccRCC tissues compared to paired normal renal tissues (200×, 400×). **e**: Kaplan–Meier analyses of the correlations between FOXO3 expression and overall survival of 530 ccRCC patients from TCGA KIRC dataset. Log-rank test was used to calculate *p* values

To investigated the potential mechanism for URRCC upregulation in ccRCC cells, bioinformatics analysis of the promoter region (− 1572 to − 1 bp) of URRCC was performed by online software RegRNA 2.0 (http://regrna2.mbc.nctu.edu.tw/detection.html) [36]. Notably, one FOXO3 binding element (− 176 to − 163 bp) was predicted (Additional file 13: Figure S4A). Moreover, ChIP assay confirmed the enrichment of FOXO3 on the predicted region (− 176 to − 163 bp) of URRCC promoter, but not on the NC site (unpredicted, − 300 to − 280) (Fig. 6a). Meanwhile, a decrease of luciferase activity on the wild type (Wt) URRCC promoter was observed in FOXO3-overexpressed A498 cells but no change on the mutant type (Mut) URRCC promoter (Fig. 6b and c). Here, IHC also confirmed that FOXO3 expression was lower in ccRCC tissues than in normal renal tissues (Fig. 6d), which was correspond with the Kaplan–Meier survival analysis from TCGA KIRC datasets that ccRCC patients with high FOXO3 expression had longer survival times than patients with low FOXO3 levels (Log-rank test, *p* < 0.001, Fig. 6e, Additional file 14: Table S10). In addition, qRT-PCR assays showed there is a negative correlation between URRCC and FOXO3 in mRNA level in cell lines (Additional file 13: Figure S4B) and knockdown of FOXO3 could enhance the mRNA expression of URRCC and EGFL7, while over-expression of FOXO3 could down-regulate the mRNA expression of URRCC and EGFL7 (Additional file 13: Figure S4C-F). Given that FOXO3 affects anti-apoptotic signal, we tried to explore the effect of URRCC on cell apoptosis in A498 cells. As figures shown, knocked-down URRCC dramatically induced the apoptosis of A498 cells, while overexpressed URRCC decreased apoptosis of A498 cells (Additional file 13: Figure S4G and S4H). Overall, these data suggested that FOXO3 silenced URRCC transcription by binding to its specific promoter in ccRCC.

## Discussion

**Fig. 7:**
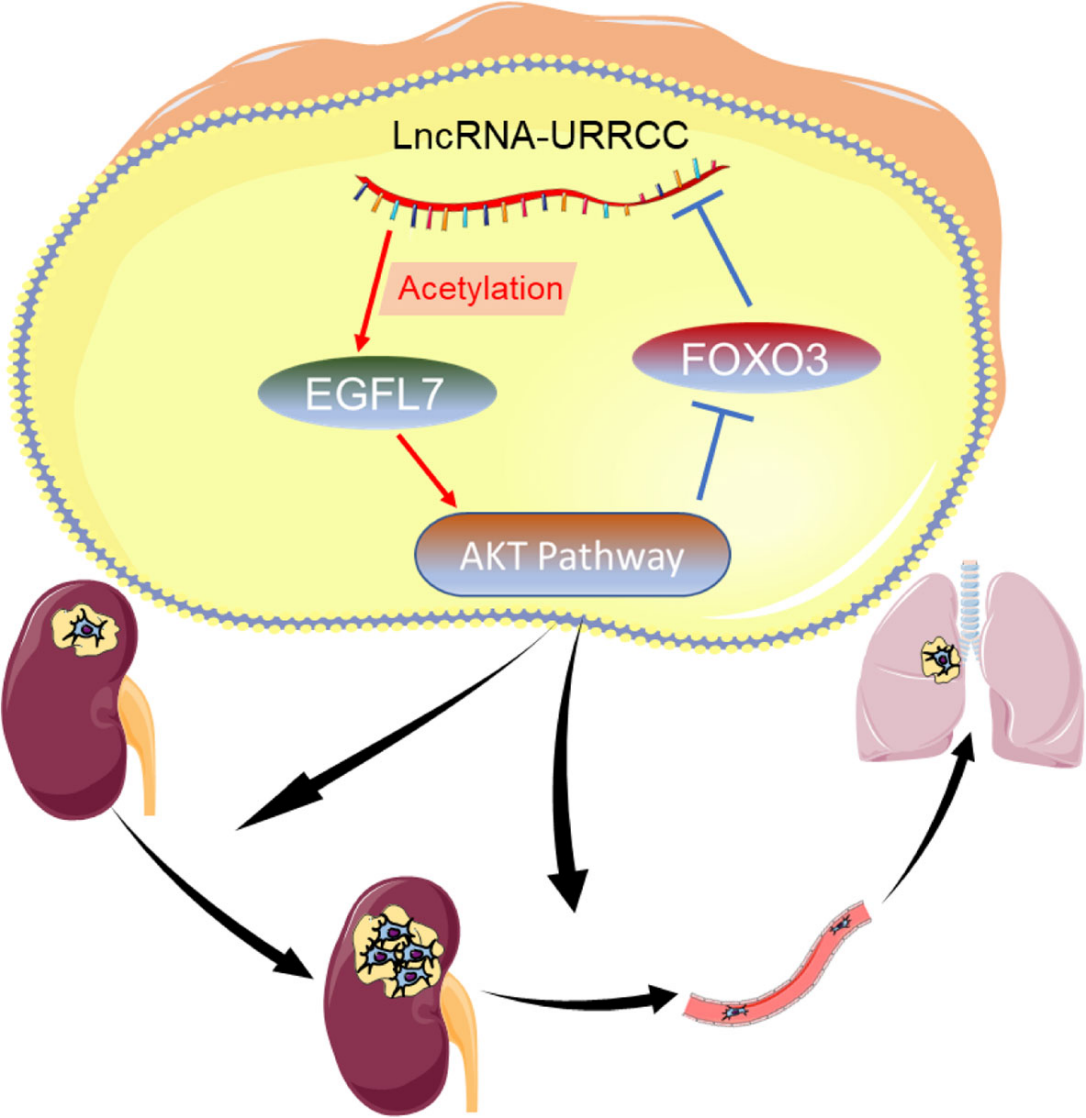
A Schematic Diagram of LncRNA-URRCC-Based Signaling Pathway in ccRCC cells proliferation and invasion. LncRNA-URRCC upregulates AKT signaling by directly targeting EGFL7 via mediating histone H3 acetylation of EGFL7 promoter, and then inducing cell growth and invasion. AKT signaling downstream FOXO3 in turn downregulates URRCC by directly binding to its promoter

In this study, we validated a new identified lncRNA-URRCC serving as a tumor-inducer in renal cancer progression through a combined bioinformatics and experimental approach. Transcriptional level of URRCC in ccRCC tissues was substantially induced compared to that in surrounding non-tumor tissues. In addition, URRCC higher levels were associated with tumor stage and invasion and inversely correlated with poor prognosis in RCC patients. URRCC enhanced RCC cell proliferation and invasion by increasing the expression of EGFL7 through regulating histone H3 acetylation of EGFL7 promoter. In addition, URRCC expression could be inhibited by transcription factor FOXO3, which led to form a regulatory circuit involving URRCC, EGFL7, P-AKT, FOXO3 (Fig. 7). All these data effectively supported that URRCC, acting as a potential prognostic biomarker in ccRCC, promoted ccRCC tumorigenesis, in keeping with the notion that lncRNAs played a pivotal role in the field of tumor initiation and development.

Intriguingly, BX649059, named as URRCC in our study, was previously discovered to be downregulated in colorectal cancer and required for obvious proliferation and invasion inhibition in colorectal cancer cells [37], contradiction our conclusion that the transcriptional level of URRCC was higher in ccRCC samples than in normal renal tissues and functioned as an onco-lncRNA to elevate cell growth and metastasis. The opposite result above suggested that URRCC might be a tissue-specific non-coding RNA in human various tissues and its expression associated with cancer development. Its clinical significance in other human cancers was yet to be elucidated. On the other hand, the expression of lncRNA was involved in clinical significance of tumor outcomes, as diagnostic or prognostic biomarkers [15, 38–40]. However, the clinical biomarker of lncRNA in the development of RCC remained to be elucidated. In the present study, we observed a negative relationship between URRCC expression and patient overall survival outcome. Statistical analysis further attested that URRCC might be an effective biomarker for helping to identify the prognosis of patients with ccRCC.

Accumulating evidence shows that lncRNAs could function as ceRNAs to sequester miRNAs, leading to the up-regulation of corresponding miRNA-targeted genes, such as lncRNA-ATB [14], or serve as molecular scaffolds to facilitate multiple proteins interaction, such as lncRNA-GClnc1 [41]. Interestingly, lncRNA that mainly located in cytoplasm also can regulate mRNA through non-ceRNA mode and lncRNA has rarely been reported to active genes by epigenetic modification [14, 25]. According to this, we sought to explore the mechanisms between lncRNA and mRNA and found that lncRNA-URRCC could enhance the expression of EGFL7 through mediating histone H3 acetylation of EGFL7 promoter. This is different from the two common mechanisms of lncRNAs above and the deeper molecular mechanism might include the interaction between URRCC and enzymes of epigenetic modification, which is worth going deep into research in the future.

The differential expression of EGFL7 in several cancers was associated with epigenetic modification, involving malignant pleural mesothelioma, gastric cancer, and esophageal squamous cell carcinoma [24, 25, 42]. Here, we identified that knocked down URRCC potently abrogated EGFL7 expression and blunted cell growth and invasion while overexpressed URRCC markedly elevated EGFL7 expression and enhanced cell growth and invasion. Mechanistically, URRCC could crosstalk with EGFL7 via mediating histone H3 acetylation of EGFL7 promoter, which is consistent with the previously report [25, 43, 44]. In addition, EGFL7 was reported to active multiply signaling pathways in human tumors [43, 45–48]. In in vitro system, in vivo system and clinical specimen, we substantiated the upregulated URRCC was potent to promote EGFL7 expression, enhancing AKT pathway, and then contribute to tumorigenesis.

Transcription factor FOXO3 is a member of the forkhead family and its activity is regulated by AKT signaling pathway [34, 35]. In our study, URRCC promoted the phosphorylation of AKT and lead to the inhibition of FOXO3. FOXO3 deregulation plays essential roles in the development of cancer and thus FOXO3 has been classified as a tumor suppressor [49]. Previous studies have demonstrated that FOXO3 could repress lncRNA and be promoted by circular RNA or Foxo3 pseudogene [33, 50]. Interestingly, our ChIP and luciferase assays showed that FOXO3 could depress URRCC expression through directly binding to URRCC promoter. Moreover, recent clinical studies have shown that FOXO3 is a good prognostic marker in some cancers [51–53]. Additionally, Kaplan–Meier survival analysis of FOXO3 in our work also could support this notion. Taken together, these results suggested that FOXO3 is an important biological factor and could be a potential target for cancer therapy.

In summary, our research reveals that URRCC functions as a tumor-inducer to control the ccRCC progression via modulating URRCC/EGFL7/P-AKT/FOXO3 positive feedback loop and cause cascading effects in ccRCC. The identification and validation of this study is a critical component of research and clinical management of ccRCC. Targeting this newly identified lncRNA-URRCC might help us to broaden the known strategy for ccRCC treatment.

## Additional files

Additional file 1: **Table S1.** Primers used for real-time PCR, vector construction, and ChIP. (XLSX 11 kb)

Additional file 2: **Table S2.** Sequences of oligonucleotides used in this study. (XLSX 10 kb)

Additional file 3: **Figure S1.** A: Flow chart of selecting dysregulated lncRNAs from two public RCC Dataset (GSE46699 and GSE53757). B and C: Heatmaps of dysregulated lncRNAs from GSE53757 (B) and GSE46699 (C). D: Comparison of AK026225 and AK055783 expression in 20 paired renal cancer tissues and adjacent non-cancer tissues via qRT-PCR. E: The chromosome location of URRCC via using ensemble software. F: The nucleotide sequence of full-length human URRCC. G: The location of URRCC predicted by lncLocator (http://www.csbio.sjtu.edu.cn/bioinf/lncLocator/). (ZIP 987 kb)

Additional file 4: **Table S3.** BX649059 expression in ccRCC and normal renal samples from TCGA KIRC dataset. (XLS 27 kb)

Additional file 5: **Table S4.** Clinical characteristics of 116 ccRCC patients according to URRCC expression levels. (XLSX 14 kb)

Additional file 6: **Table S5.** Univariate and Multivariate Cox Regression Analyses of URRCC. (XLSX 11 kb)

Additional file 7 **Figure S2.** A and B: qRT-PCR assays for the URRCC mRNA level in 786-O and OSRC-2 cells after transfection of sh-control, sh-URRCC-1, and sh-URRCC-2. C: MTT assays after transfection of sh-URRCC compared with sh-control in 786-O cells. D: Representative images and the numbers of invasive cells per high-power field reduced by the transfection of sh-URRCC in 786-O compared to sh-control groups. (PDF 96 kb)

Additional file 8: **Figure S3.** A: Heatmaps of dysregulated mRNAs expression between sh-control and sh-URRCC groups in the treated A498 cells. B: Volcano plots of the differentially expressed mRNAs. Green and red spots represent *p* value less than 0.05. Red spots represent fold change more than 2.0. Green spots represent fold change less than 0.5. C: JMY mRNA level in ccRCC tissues compared with normal renal tissues from TCGA KIRC dataset. D: Kaplan–Meier analyses of the correlations between URRCC expression and overall survival of 530 ccRCC patients from TCGA KIRC dataset. Log-rank test was used to calculate *p* values. E: Correlation between URRCC and EGFL7 in mRNA level in cell lines. F: mRNA level of EGFL7 in different treatment groups. G and H: ChIP analyses of A498 and OSRC-2 cells treated with sh-control, sh-URRCC or sh-URRCC+TSA(100 nM) were conducted on the EGFL7 promoter regions using anti-acetyl-histone H3 and anti-acetyl-histone H4. Enrichment was determined relative to input controls. I: Representative Ki67 IHC staining of xenograft tumors from sh-control, sh-URRCC, mock, and oe-URRCC groups (200×, 400×). (ZIP 220 kb)

Additional file 9: **Table S6.** Small mRNA array data. (XLSX 147 kb)

Additional file 10: **Table S7.** JMY expression in ccRCC and normal renal samples from TCGA KIRC dataset. (XLS 27 kb)

Additional file 11: **Table S8.** JMY expression and paitients’ survival time from TCGA KIRC dataset. (XLS 19 kb)

Additional file 12: **Table S9.** EGFL7 expression in ccRCC and normal renal samples from TCGA KIRC dataset. (XLS 26 kb)

Additional file 13: **Figure S4.** A: Bioinformatics analysis of potential FOXO3 binding site on URRCC promoter by online software RegRNA 2.0. B: Correlation between URRCC and FOXO3 in mRNA level in cell lines. C and D: The mRNA level of URRCC and EGFL7 were detected by qRT-PCR in A498 and OSRC-2 cells after transfection with si-NC or si-FOXO3. E and F: The mRNA level of URRCC and EGFL7 were detected by qRT-PCR in A498 and OSRC-2 cells after transfection with NC or oe-FOXO3. G and H: Cell apoptosis assays by flow cytometry in A498 cell lines. (ZIP 209 kb)

Additional file 14: **Table S10.** FOXO3 expression and paitients' survival time from TCGA KIRC dataset. (XLS 19 kb)

## Abbreviations

ccRCC: Clear cell renal cell carcinoma
ceRNA: Competing endogenous RNA
ChIP: Chromatin immunoprecipitation
FISH: Fluorescent in situ hybridization
GEO: Gene Expression Omnibus
IHC: Immunohistochemistry
IVIS: In vivo imaging systems
KIRC: Kidney renal clear cell carcinoma
lncRNA: Long noncoding RNA
qRT-PCR: Quantitative real-time PCR
RCC: Renal cell carcinoma
shRNA: Short hairpin RNA
siRNA: Short interfering RNA
TCGA: The Cancer Genome Atlas
TSS: Transcriptional start site
URRCC: Up-regulation in clear cell renal cell carcinoma
WB: Western blot
