# Supplementary material for: A positive feed-forward loop between LncRNA-URRCC and EGFL7/P-AKT/FOXO3 signaling promotes proliferation and metastasis of clear cell renal cell carcinoma
Source: Mol Cancer. 2019 Apr 5;18:81. doi: 10.1186/s12943-019-0998-y (PMC6449923; doi:10.1186/s12943-019-0998-y)
Supplement: Supplementary file 3 — Figure S1. A: Flow chart of selecting dysregulated lncRNAs from two public RCC Dataset (GSE46699 and GSE53757). B and C: Heatmaps of dysregulated lncRNAs from GSE53757 (B) and GSE46699 (C). D: Comparison of AK026225 and AK055783 expression in 20 paired renal cancer tissues and adjacent non-cancer tissues via qRT-PCR. E: The chromosome location of URRCC via using ensemble software. F: The nucleotide sequence of full-length human URRCC. G: The location of URRCC predicted by lncLocator (http://www.csbio.sjtu.edu.cn/bioinf/lncLocator/). (ZIP 987 kb) [file 12943_2019_998_MOESM3_ESM.zip › Supplementary Figure-R 1_2.pdf]

FigS1.

F

```
   1 gtacctgatt cgcctgccag aacacctcag cctcctgaaa tgaatccttt gtcagcagtt
  61 aacatgtttc agaaacaaaa ttcaaaaccc agcgtgccag ttagtattcc aaaaagcaaa
 121 gaaaaacagg gacgtccacc aggtgcattg gtgccagcat cttcactgaa aggaggtaat
 181 ctgggctcta tgtcagtccg ttctaaattg ccaaattctc cagcagcatc ttctcatccc
 241 aagctcaagt cttcaaaagg cataacgaag aaaccgcagg ctccttcaaa caatgcatca
 301 tcttcacttg cttcattaaa tccagtaggt aaaaacactt cttcaccagc tttaccaaga
 361 actgcacctt gtatatctga gtcaccgaga aaatgtattt catcccccaa tacccccaag
 421 gccaaggtta ttccagccca gaattcagca gatctgcccg agtccacact tttgccaaat
 481 aagtgttcag gaaaaactca acctaagtat ttgaaacata atcatatttc ttccagagat
 541 aatgcagtat ctcacttagc tgcacattca aattcatcct caaaatgtcc caagctgcct
 601 aaagcaaata tacctgtaag acctaaacct tctttccagt cctctgcaaa aatgacaaaa
 661 accagttcca aaaccatagc cacgggtcta ggaacacagt ctcaaccatc cgatggagcc
 721 ccacaagcaa agccagtccc agcacagaaa cttaaatcgg ccttgaattt aaatcagcca
 781 gtttctgtgt cctcagtttc tcctgtaaaa gccacacaga aatcaaaaga taagaatata
 841 gtttcagcta ccaaaaagca gcctcagaat aaaagtgcat ttcagaagac aggacccagc
 901 tccttgaagt ctcctggccg taccccactg tccatcgtga gcctacccca gtcttctacc
 961 aaaacacaaa ctgcaccgaa gtcagcacag actgtcgcta agagccagca ttcaactaaa
1021 gggcctccca gaagtggtaa aaccccagct tcaatcagga aaccaccctc atctgttaag
1081 gatgcagata gtggagataa aaaacctact gcaaagaaaa aggaagatga tgaccattat
1141 tttgtcatga ctggaagtaa gaaacctaga aaataaatac atactcatta taaaaaaaga
1201 gaaaaggaag aatgaatgtg ttagcttcac atcttaaaag tttctcctat ttgtgtctgt
1261 ctaaataggt gctgacacta aggatagtga ggatggaggc tgggatgagg aaagggttca
1321 tcagaattca catatctgaa ttcactggaa agagcccttc tgaagcaaac agttgtaaaa
1381 tcactgcaag gtttttatta ataatagaca tgtatatgat tttcagtcta tagcatcttt
1441 gttaacatct gcctttttgca ggaaatgtaa aagttattta acactacaag aattttaaca
1501 atagttgctc tatttttgaa tatgtattaa atatggagtt catatacctg ctaacatcaa
1561 cggtggtgct cttactatta gttaattgca ttttggttaa aaaaaaaaaa gcaacagttt
1621 ggcacttgtc ctacaaaagg cacctaattt aattttctga tcaggattgc ctgatccaac
1681 agtgctaagt catggctgct gctgactagc ttggcattat tctgtgttag gtagaattct
1741 tattatttat tttttaagc tttccaaatt ggaaggaact gattgtttca tgtggcttat
1801 atttacattg gtaatatttt gtcaccaata tttttggtta aaaaaaatcc aacaaattaa
1861 cttactgaaa tataaacaaa ttttgtaaac aatttttat attatctata aaaacgtaga
1921 caccttatgt ttcacatgtt gtgcaatgtg acaggggaag ctgatttagt agcttttagc
1981 atattaaaaa taattttta taatgtaatt tcctgtgagt gcagacctga cattttacat
2041 taaaataatg tgaaacatca gaattatgtt ttaacaactt taaaattaag atgatgttaa
2101 aataatttta gagttatgct atgtaaaaat tctatcatga aattattttt ctctagatag
2161 cacaatacca attttaatta atttcttcca attaggttac ttttctttaa taaagttatg
2221 ctgccttcag ttttccaatg gcaagtagac aggatatgtt caaggttttc tgcactgtag
2281 gcacagtctc tcaagcatat cctgatcatg taatgactgc ataaactcca tcaacctaag
2341 gtgatacttg taaataattt atttttaagg gatggtgact ttaaaaatta ttaatgaact
2401 ttgagaagtt ttaagagtgc ttttaaaact tcacagtatt gccaattatc ttaggttatt
2461 cagtattcag gtttgtgttt ctctgtttta aactaaaatg tgttttctga agaaaaaaat
2521 aatagtttac acaaatgtac aatcatagaa taagcatttt aagctggcga ctagtgttct
2581 atagattaca aagcaagaaa actttctatg aagataaatg accttttgcc tgaagagtac
2641 agataaaatc aaagatgtgt gcaagctagt ttttggaaga agtgatgctt ctcttcttta
2701 aagagacagt caccaaatac ttggtttaac tcgactattg acttgggcat tgagagagat
2761 gatatataca tctttggaaa gtgaagtcaa tgttcaagag gtgatagaag ctttactttt
2821 tagtgatcag aaatatttag tgcatctttt cagacaggaa gaattttatc atcaagtatt
2881 cccttataaa accaagtaac gcttctttat cagtaacttt tagaacttaa aagaaagcaa
2941 aaagtaaatg gaattgtagg caatttatga atcctagtag attttacaat atgtaattta
3001 tgttgtttac agtatataaa cactaagttt tgtgttaaat gtgatcagga ataaaagtat
3061 cccacaggca tctgacacaa attccagaat tagccaaaga attgtttatt tgaggccagg
3121 caatcccagc attttgggaa gccaagttgg gctgatctcg aactcctgac ctcaggtgat
3181 ccacctgcct cagcctccca aagcgctggg attacaggca tgagccatca cacccagcga
3241 aaagttttgt ttgaataaac aatatccgaa agacaattag tttcttcaga tgtgttttga
3301 aattctccta aagagctagt gtttctattc attttcacaa tttaaaaaca gctcttaaca
3361 ttgctgaagt tgggagaact ttccatctct tcttaataac agtgcaagat tttgtaaatt
3421 ctttttttgtg tttaatgttt aataaaacga gtattaagct taaattactg aagtacctgg
3481 gagaagtaat gatgtgtact ttcaaaaaaa tggaaaatgc ttttatttta ttttctataa
3541 tttgttaaca tgatatgtaa aattaaactt cggagcacaa tgaaatgccg attattttta
3601 ccttgtttgg gcttaaagta ggtatttaag gtttatgtgt tcaaaatgcc ttggtaaatt
3661 ggatgacctc taactttact gtccatatgg agtttgtcat tctttatgga taagagaact
3721 taaggaaaag ttactgtttt tcttcagtct ttttatatct atctgattta aaatctgtta
3781 ctttattaaa aggcttcaac aacaggttgt taggatgtag tcttacatcc aggttcacat
3841 aataacccca tttgaatcca aatttgtgta tattttctta tgccagcagt atttgtatcc
3901 aattttaact taggtttgtt ttcttgagta ttaaaattta aacatataaa aaaaaaaaaa
3961 aaaaaaa
```

G

------------ lncLocator Prediction Result ------------

Subcellular locations	score
Cytoplasm	0.879770153137
Nucleus	0.057460902964
Ribosome	0.0200502799862
Cytosol	0.039617910722
Exosome	0.00310075319078

Predicted location
Cytoplasm
