# Supplementary figures and images for: A positive feed-forward loop between LncRNA-URRCC and EGFL7/P-AKT/FOXO3 signaling promotes proliferation and metastasis of clear cell renal cell carcinoma
Source: Mol Cancer. 2019 Apr 5;18:81. doi: 10.1186/s12943-019-0998-y (PMC6449923; doi:10.1186/s12943-019-0998-y)

FigS1.

A

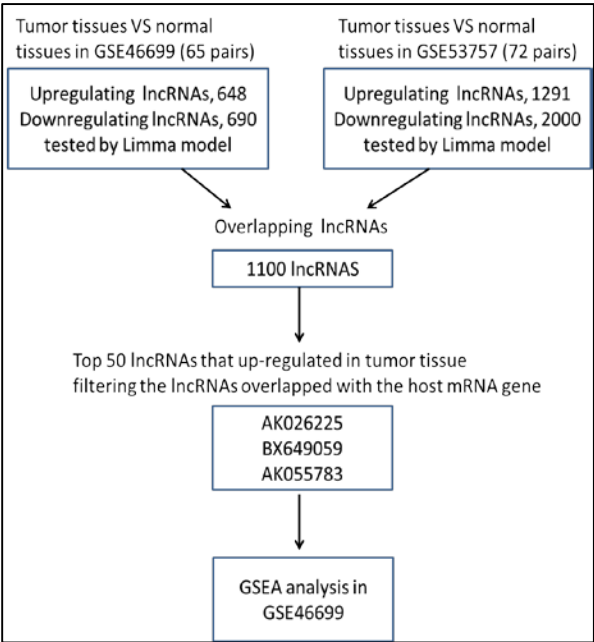

B

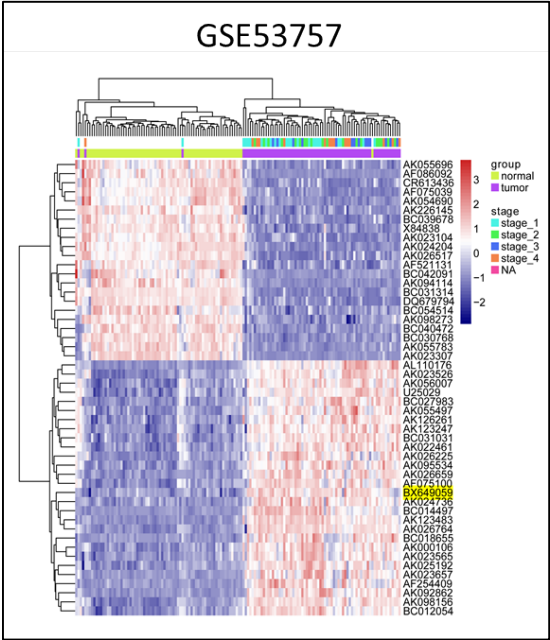

C

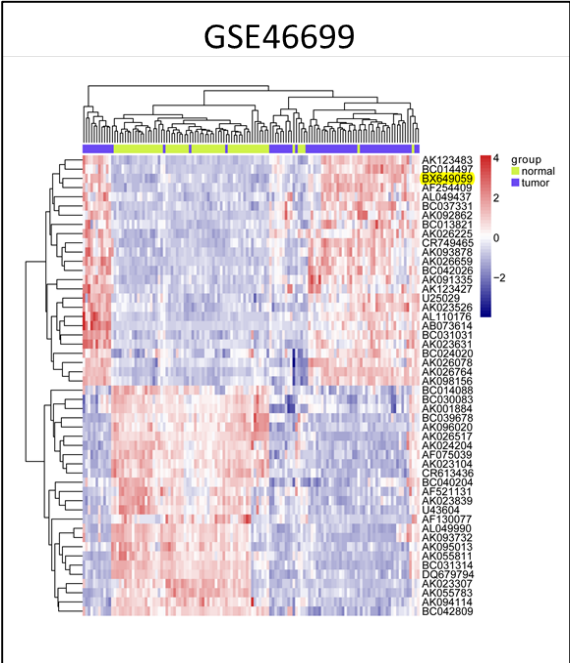

D

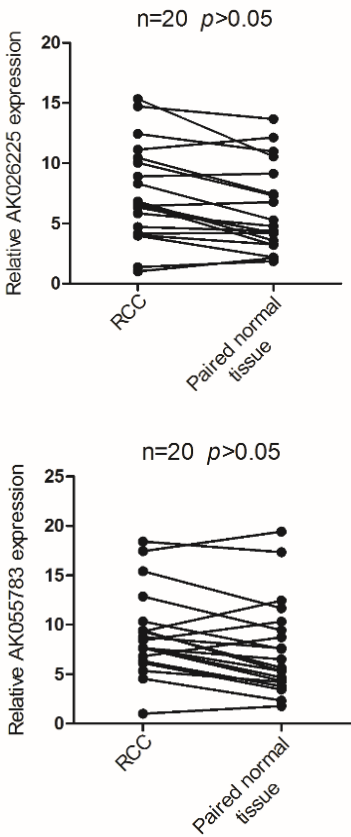

E

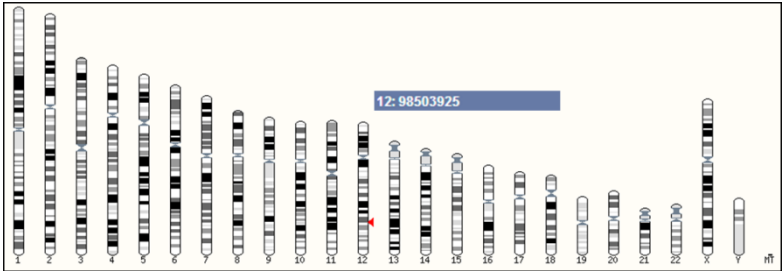

Supplement: Supplementary file 3 — Figure S1. A: Flow chart of selecting dysregulated lncRNAs from two public RCC Dataset (GSE46699 and GSE53757). B and C: Heatmaps of dysregulated lncRNAs from GSE53757 (B) and GSE46699 (C). D: Comparison of AK026225 and AK055783 expression in 20 paired renal cancer tissues and adjacent non-cancer tissues via qRT-PCR. E: The chromosome location of URRCC via using ensemble software. F: The nucleotide sequence of full-length human URRCC. G: The location of URRCC predicted by lncLocator (http://www.csbio.sjtu.edu.cn/bioinf/lncLocator/). (ZIP 987 kb) [file 12943_2019_998_MOESM3_ESM.zip › Supplementary Figure-R 1_1.pdf]

FigS2.

A

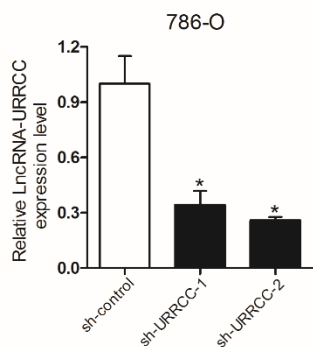

B

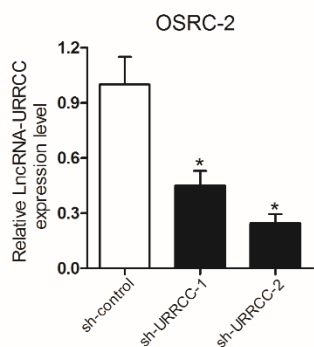

C

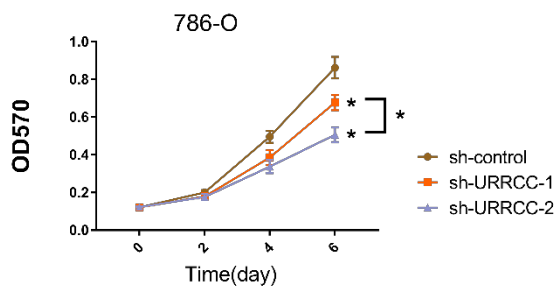

D

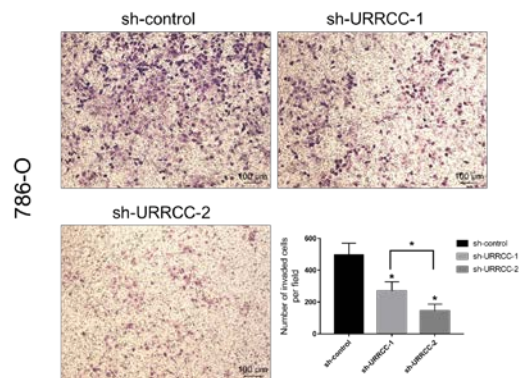

Supplement: Supplementary file 7 — Figure S2. A and B: qRT-PCR assays for the URRCC mRNA level in 786-O and OSRC-2 cells after transfection of sh-control, sh-URRCC-1, and sh-URRCC-2. C: MTT assays after transfection of sh-URRCC compared with sh-control in 786-O cells. D: Representative images and the numbers of invasive cells per high-power field reduced by the transfection of sh-URRCC in 786-O compared to sh-control groups. (PDF 96 kb) [file 12943_2019_998_MOESM7_ESM.pdf]

FigS3.

A

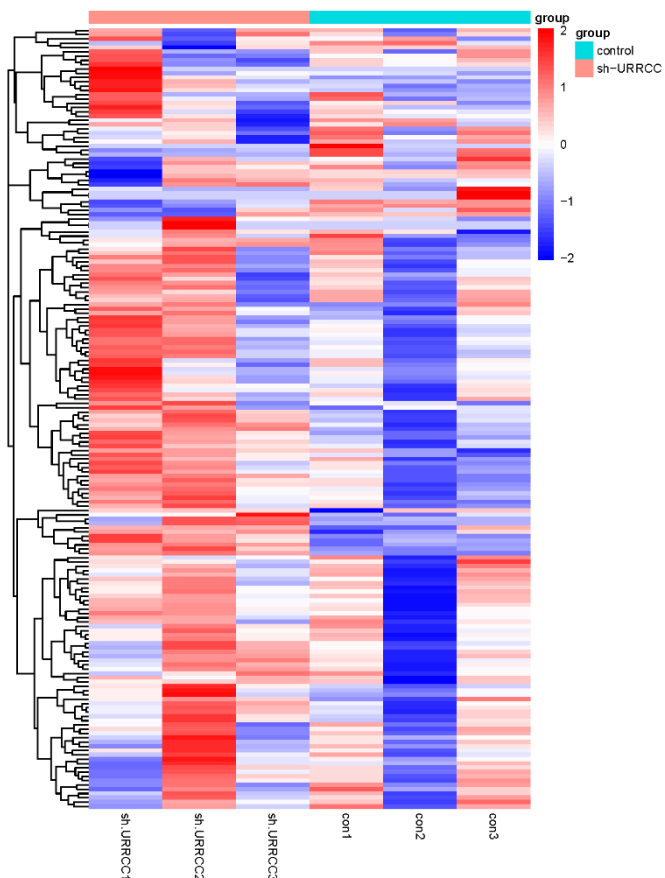

B

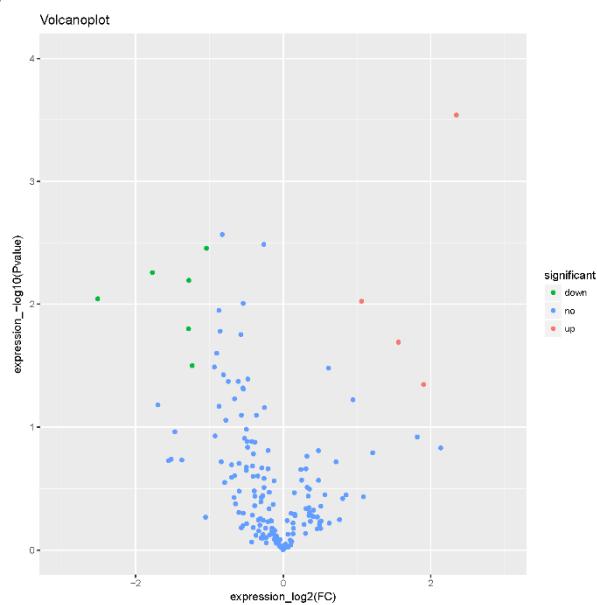

C

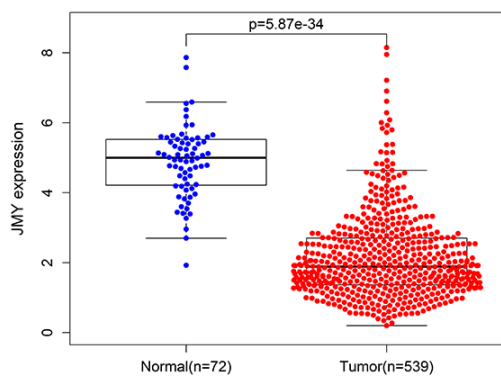

D

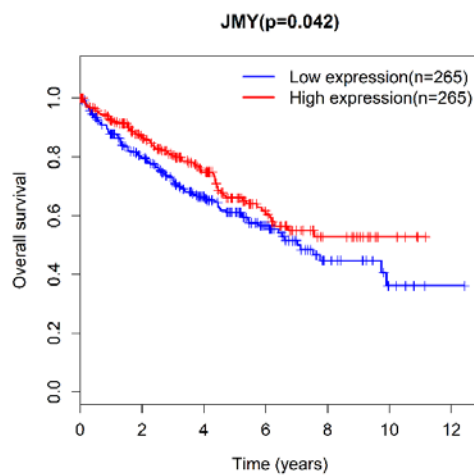

E

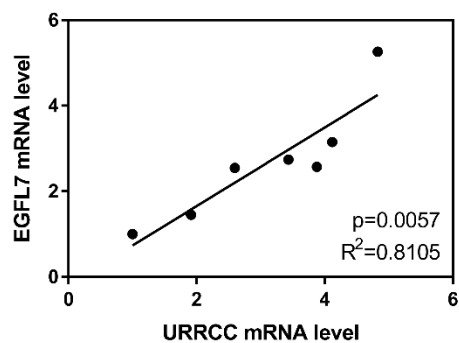

F

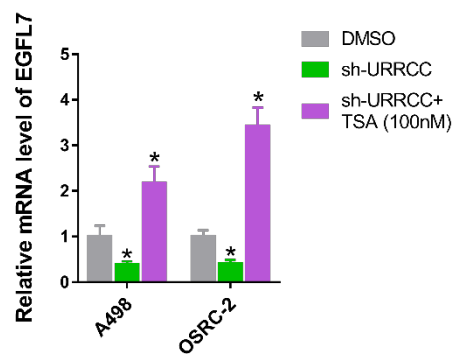

Supplement: Supplementary file 8 — Figure S3. A: Heatmaps of dysregulated mRNAs expression between sh-control and sh-URRCC groups in the treated A498 cells. B: Volcano plots of the differentially expressed mRNAs. Green and red spots represent p value less than 0.05. Red spots represent fold change more than 2.0. Green spots represent fold change less than 0.5. C: JMY mRNA level in ccRCC tissues compared with normal renal tissues from TCGA KIRC dataset. D: Kaplan–Meier analyses of the correlations between URRCC expression and overall survival of 530 ccRCC patients from TCGA KIRC dataset. Log-rank test was used to calculate p values. E: Correlation between URRCC and EGFL7 in mRNA level in cell lines. F: mRNA level of EGFL7 in different treatment groups. G and H: ChIP analyses of A498 and OSRC-2 cells treated with sh-control, sh-URRCC or sh-URRCC+TSA(100 nM) were conducted on the EGFL7 promoter regions using anti-acetyl-histone H3 and anti-acetyl-histone H4. Enrichment was determined relative to input controls. I: Representative Ki67 IHC staining of xenograft tumors from sh-control, sh-URRCC, mock, and oe-URRCC groups (200×, 400×). (ZIP 220 kb) [file 12943_2019_998_MOESM8_ESM.zip › Supplementary Figure-R 3_1.pdf]

G

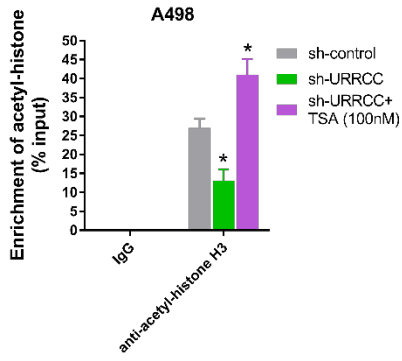

H

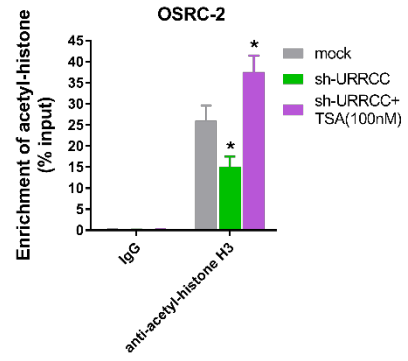

I

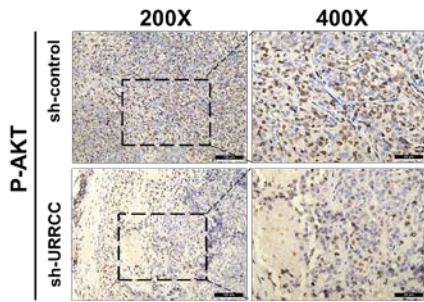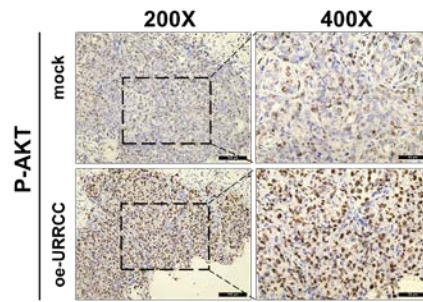

Supplement: Supplementary file 8 — Figure S3. A: Heatmaps of dysregulated mRNAs expression between sh-control and sh-URRCC groups in the treated A498 cells. B: Volcano plots of the differentially expressed mRNAs. Green and red spots represent p value less than 0.05. Red spots represent fold change more than 2.0. Green spots represent fold change less than 0.5. C: JMY mRNA level in ccRCC tissues compared with normal renal tissues from TCGA KIRC dataset. D: Kaplan–Meier analyses of the correlations between URRCC expression and overall survival of 530 ccRCC patients from TCGA KIRC dataset. Log-rank test was used to calculate p values. E: Correlation between URRCC and EGFL7 in mRNA level in cell lines. F: mRNA level of EGFL7 in different treatment groups. G and H: ChIP analyses of A498 and OSRC-2 cells treated with sh-control, sh-URRCC or sh-URRCC+TSA(100 nM) were conducted on the EGFL7 promoter regions using anti-acetyl-histone H3 and anti-acetyl-histone H4. Enrichment was determined relative to input controls. I: Representative Ki67 IHC staining of xenograft tumors from sh-control, sh-URRCC, mock, and oe-URRCC groups (200×, 400×). (ZIP 220 kb) [file 12943_2019_998_MOESM8_ESM.zip › Supplementary Figure-R 3_2.pdf]

FigS4.

A

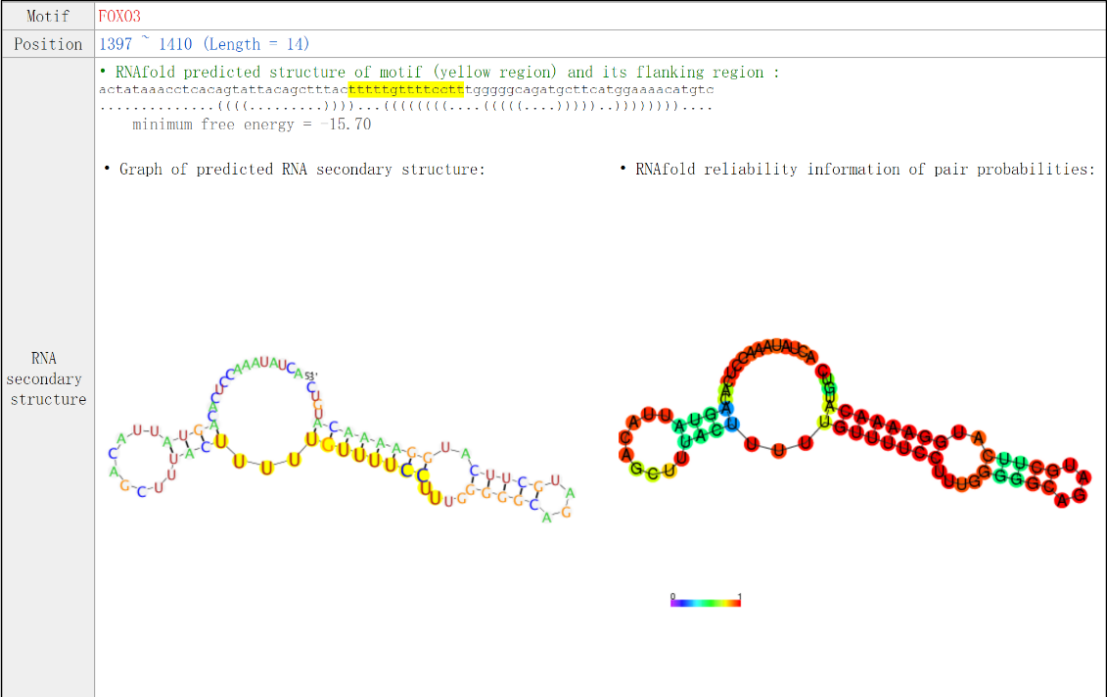

B

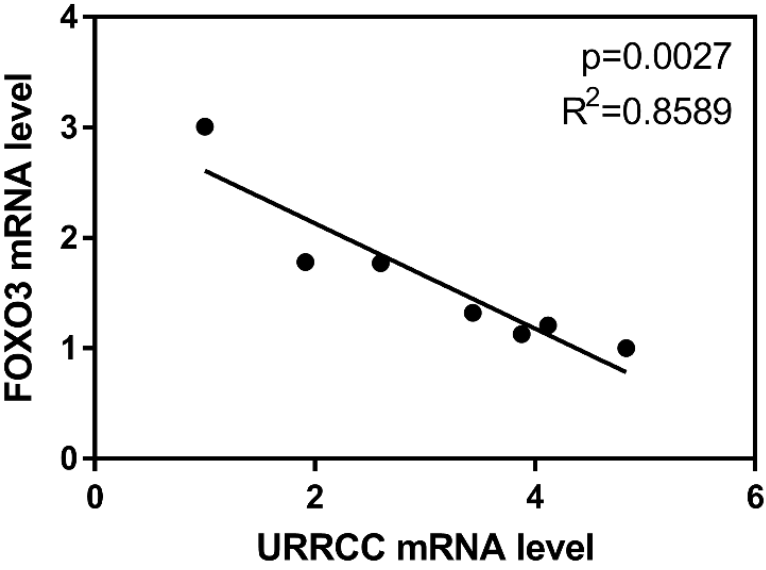

Supplement: Supplementary file 13 — Figure S4. A: Bioinformatics analysis of potential FOXO3 binding site on URRCC promoter by online software RegRNA 2.0. B: Correlation between URRCC and FOXO3 in mRNA level in cell lines. C and D: The mRNA level of URRCC and EGFL7 were detected by qRT-PCR in A498 and OSRC-2 cells after transfection with si-NC or si-FOXO3. E and F: The mRNA level of URRCC and EGFL7 were detected by qRT-PCR in A498 and OSRC-2 cells after transfection with NC or oe-FOXO3. G and H: Cell apoptosis assays by flow cytometry in A498 cell lines. (ZIP 209 kb) [file 12943_2019_998_MOESM13_ESM.zip › Supplementary Figure-R 4_1.pdf]

C

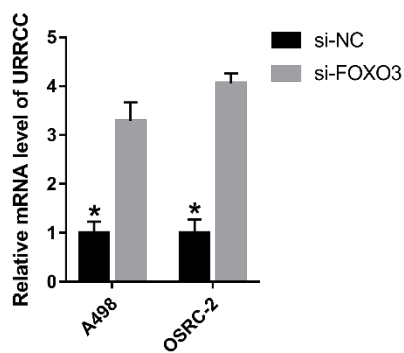

D

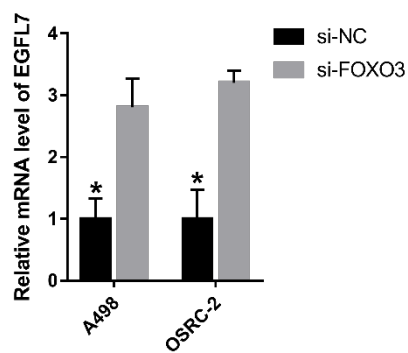

E

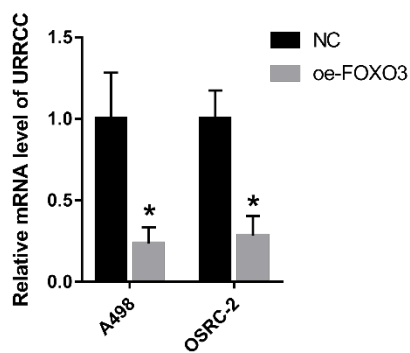

F

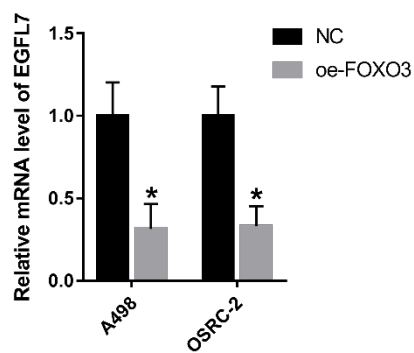

G

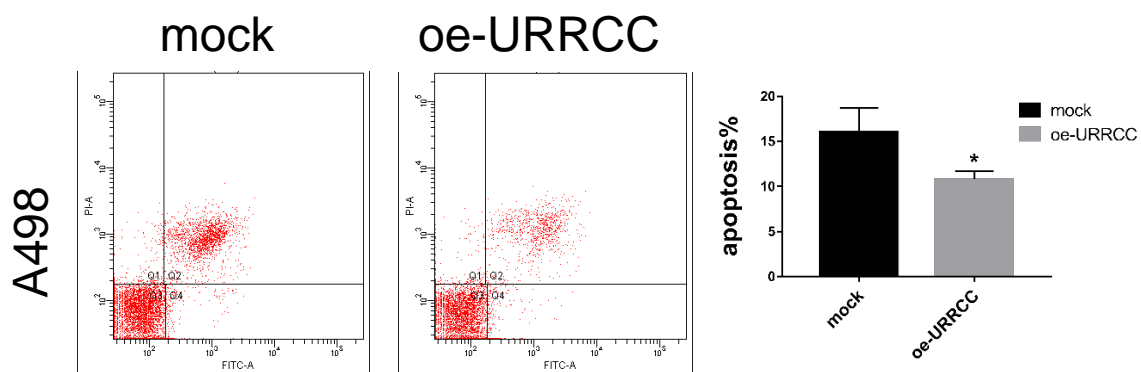

H

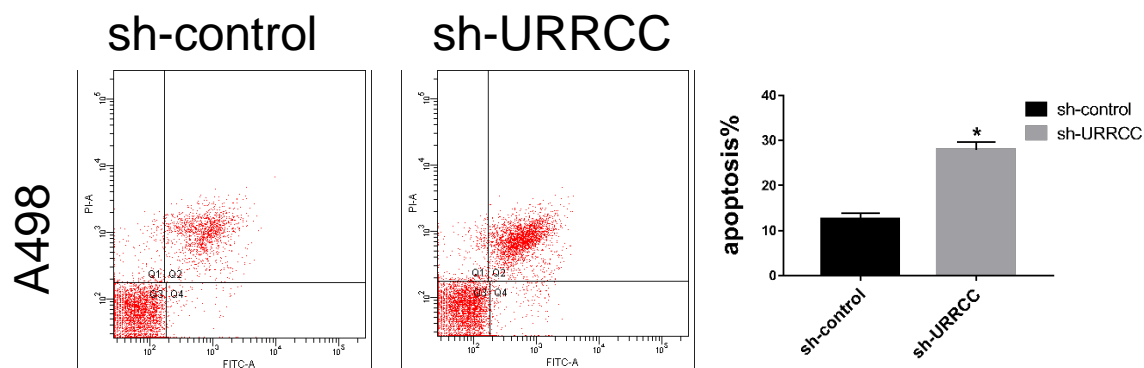

Supplement: Supplementary file 13 — Figure S4. A: Bioinformatics analysis of potential FOXO3 binding site on URRCC promoter by online software RegRNA 2.0. B: Correlation between URRCC and FOXO3 in mRNA level in cell lines. C and D: The mRNA level of URRCC and EGFL7 were detected by qRT-PCR in A498 and OSRC-2 cells after transfection with si-NC or si-FOXO3. E and F: The mRNA level of URRCC and EGFL7 were detected by qRT-PCR in A498 and OSRC-2 cells after transfection with NC or oe-FOXO3. G and H: Cell apoptosis assays by flow cytometry in A498 cell lines. (ZIP 209 kb) [file 12943_2019_998_MOESM13_ESM.zip › Supplementary Figure-R 4_2.pdf]
